# A Review of Terpenes from Marine-Derived Fungi: 2015–2019

**DOI:** 10.3390/md18060321

**Published:** 2020-06-18

**Authors:** Minghua Jiang, Zhenger Wu, Heng Guo, Lan Liu, Senhua Chen

**Affiliations:** 1 School of Marine Sciences, Sun Yat-sen University, Guangzhou 510006, China; jiangmh23@mail2.sysu.edu.cn (M.J.); wuzher@mail2.sysu.edu.cn (Z.W.); hengeguo163@163.com (H.G.); cesllan@mail.sysu.edu.cn (L.L.); 2 South China Sea Bio-Resource Exploitation and Utilization Collaborative Innovation Center, Guangzhou 510006, China; 3 Southern Laboratory of Ocean Science and Engineering (Guangdong, Zhuhai), Zhuhai 519000, China

**Keywords:** terpenoid, biological activity, chemical diversity, marine natural product, marine fungi

## Abstract

Marine-derived fungi are a significant source of pharmacologically active metabolites with interesting structural properties, especially terpenoids with biological and chemical diversity. In the past five years, there has been a tremendous increase in the rate of new terpenoids from marine-derived fungi being discovered. In this updated review, we examine the chemical structures and bioactive properties of new terpenes from marine-derived fungi, and the biodiversity of these fungi from 2015 to 2019. A total of 140 research papers describing 471 new terpenoids of six groups (monoterpenes, sesquiterpenes, diterpenes, sesterterpenes, triterpenes, and meroterpenes) from 133 marine fungal strains belonging to 34 genera were included. Among them, sesquiterpenes, meroterpenes, and diterpenes comprise the largest proportions of terpenes, and the fungi genera of *Penicillium*, *Aspergillus*, and *Trichoderma* are the dominant producers of terpenoids. The majority of the marine-derived fungi are isolated from live marine matter: marine animals and aquatic plants (including mangrove plants and algae). Moreover, many terpenoids display various bioactivities, including cytotoxicity, antibacterial activity, lethal toxicity, anti-inflammatory activity, enzyme inhibitor activity, etc. In our opinion, the chemical diversity and biological activities of these novel terpenoids will provide medical and chemical researchers with a plenty variety of promising lead compounds for the development of marine drugs.

## 1. Introduction

Marine-derived fungi are a significant source of pharmacologically active metabolites with interesting structural properties [1,2,3,4,5]. Terpenoids, which possess chemical structure diversity [6], are important secondary metabolites of marine-derived fungi and have diverse bioactivities [7,8], such as cytotoxic, antibacterial, antifungal, antiviral, anti-inflammatory, and enzyme inhibitor activities. Two past reviews have covered the isolation, chemical structures, and biological activities of terpenoids from marine-derived fungi—one published by Rainer Ebel [7] in 2011 and another by Peter Proksch [8] in 2015. The field of marine natural products is currently in a golden age of microbially derived compound discovery [9]; there has been a tremendous increase in the rate of new terpenoids from marine-derived fungi being discovered in the last five years. Hence, an updated review is needed, covering the literature published from 2015 to 2019. In this review, we focus not only on the diversity of the chemical structures and bioactive properties of the new terpenes isolated from marine-derived fungi during the last five years but also on the biodiversity of these fungi. A total of 140 research papers describing 471 new terpenoids from marine fungi were included in this analysis. Steroids and simple isopentenyl (C5)-substituted alkaloids, polyketides, peptides, and shikimates, and their derivates, were excluded from this review.

## 2. The Characteristics of Terpenoids from Marine Fungi

**Figure 1:**
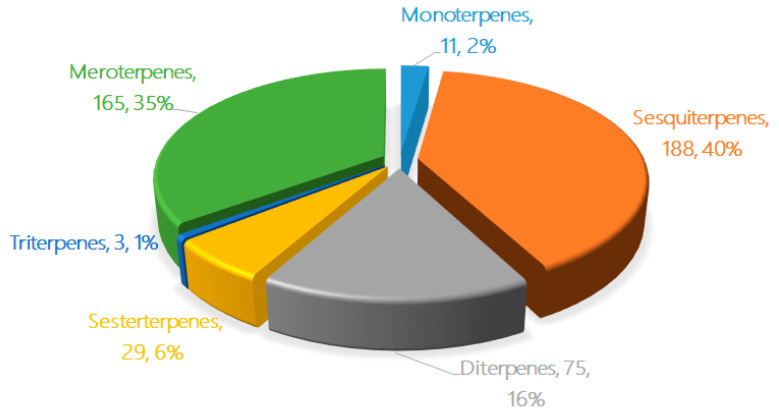
The proportions of different terpenes from marine fungi discovered in the last five years.

The new terpenoids from marine fungi can be divided into six groups based on their chemical structures and biogenetic pathways: monoterpenes, sesquiterpenes, diterpenes, sesterterpenes, triterpenes, and meroterpenes. The pie chart in Figure 1 details the proportions of the different terpenes from marine fungi discovered in the last five years. Sesquiterpenes (188, 40%), meroterpenes (165, 35%), and diterpenes (75, 16%) comprise the largest proportions of terpenes from marine fungi, followed by sesterterpenes (29, 6%), monoterpenes (11, 2%), and triterpenes (3, 1%). The number of new terpenoids found in marine fungi increased rapidly from 2010–2014 [8] to 2015–2019 (on average, an increase from 40 discoveries a year to 95 a year).

**Figure 2:**
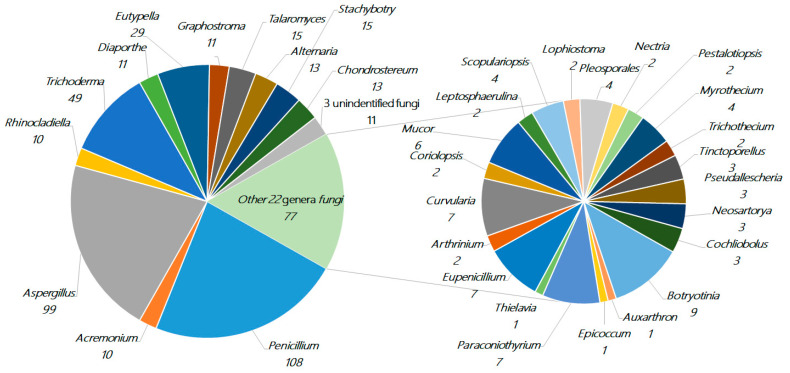
The terpenoids from marine fungi in this review divided by the origin of the genus.

About 471 new terpenes were derived from a diverse range of marine fungi (127 strains, including three strains of unidentified fungi) belonging to 34 genera (*Acremonium*, *Alternaria*, *Arthrinium*, *Aspergillus*, *Auxarthron*, *Botryotinia*, *Chondrostereum*, *Cochliobolus*, *Coriolopsis*, *Curvularia*, *Diaporthe*, *Epicoccum*, *Eupenicillium*, *Eutypella*, *Graphostroma*, *Leptosphaerulina*, *Lophiostoma*, *Mucor*, *Myrothecium*, *Nectria*, *Neosartorya*, *Paraconiothyrium*, *Penicillium*, *Pestalotiopsis*, *Pleosporales*, *Pseudallescheria*, *Rhinocladiella*, *Scopulariopsis*, *Stachybotrys*, *Talaromyces*, *Thielavia*, *Tinctoporellus*, *Trichoderma*, and *Trichothecium*). *Penicillium* (23%, 108), *Aspergillus* (21%, 99), and *Trichoderma* (10%, 49) each represent more than 10% of the total and are the dominant producers of terpenoids, whose amounts together comprise more than half of the total. There are nine genera of fungi in the range of 2%–6% (10–29 compounds), including *Eutypella* (6.0%, 29), *Talaromyces* (3.2%, 15), *Stachybotrys* (3.2%, 15), *Alternaria* (2.8%, 13), *Chondrostereum* (2.8%, 13), *Diaporthe* (2.3%, 11), *Graphostroma* (2.3%, 11), *Rhinocladiella* (2.1%, 10), and *Acremonium* (2.1%, 10). The remaining 22 genera of fungi together make up approximately 19%, while each genus comprises less than 2% (≤9 compounds), as shown in Figure 2.

**Figure 3:**
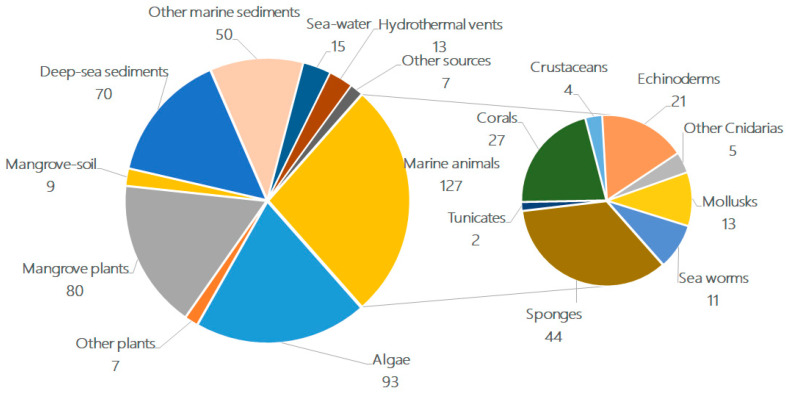
The terpenoids from marine fungi were divided by their sources (habitats); 471 terpenoids were isolated from 127 species of fungi in 127 habitats.

When examining the habitats/sources of these terpenoid-rich marine fungi (Figure 3), were found that 33% of the compounds were isolated from marine environments (i.e., deep-sea sediments (15%)) and other marine sediments (shallow sea or coast, 11%), while the remaining compounds were obtained from living matter, such as marine animals (27%) and aquatic plants (including mangrove plants) (38%). Within the individual groups, algae (20%), mangrove habitats (19%), deep-sea sediments (15%), other marine sediments (11%), sponges (9%), coral (6%), and echinoderms (4%) were the most predominant sources of fungi. A newly emerging source is the extreme environment, i.e., deep-sea sediments (15%) and hydrothermal vents (3%), which can produce structurally unique metabolites.

**Figure 4:**
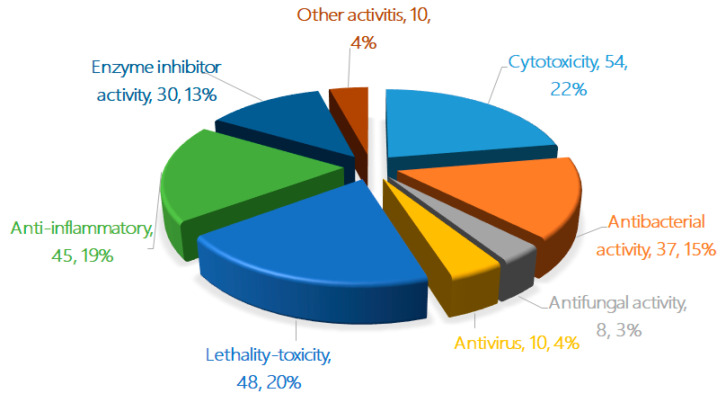
The percentage represents the proportion of one activity compared to the whole occurrence of activities of bioactive terpenoids from marine fungi.

**Figure 5:**
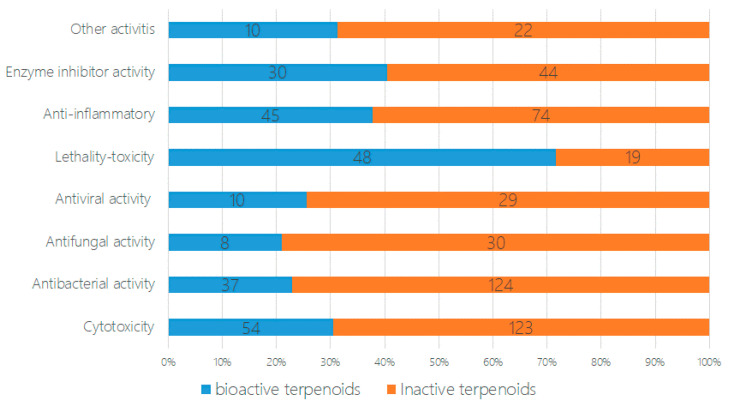
The number of bioactive terpenoids and inactive terpenoids from marine fungi evaluated by eight classes of bioactivity modes.

In the bioassays of the 471 new terpenoids from marine fungi, most of the isolated novel terpenoids were evaluated as having one or more bioactivity (Figure 4 and Figure 5). An equivalent of 30% of the terpenoids displayed biological activities, including cytotoxicity, antibacterial activity, antifungal activity, antiviral activity, lethal toxicity activity, anti-inflammatory activity, enzyme inhibitor activity, and other activities (242 active/707 test of terpenoids). In total, 22% of the compounds displayed cytotoxicity (up to 54 compounds), followed by lethal toxicity (20%, 48), anti-inflammatory (19%, 45), antibacterial (15%, 37), protease enzyme inhibition (13%, 30), antiviral (4%, 10), and antifungal (3%, 8) activities (Figure 4). When comparing the special activities of the new terpenoids, the lethal toxicity (mainly including the toxicity of marine phytoplankton, marine zooplankton, and plant seedlings) (70.6%, 20/67), enzyme inhibitor activity (40.5%, 44/74), anti-inflammatory activity (37.8%, 45/119), and cytotoxicity (30.5%, 54/177) of each terpenoid should be given more attention in the search for new drug leads (Figure 5).

## 3. Isolation, Structure, and Bioactivities of Terpenoids from Marine Fungi

### 3.1. Monoterpenes

**Figure 6:**
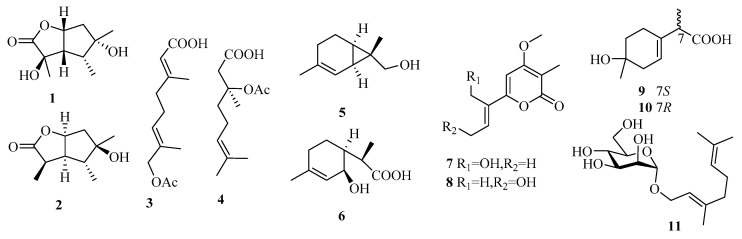
Chemical structures of monoterpenes (**1**–**11**).

While monoterpenes were rarely isolated from fungi in the past 15 years, there has been a significant increase in the number of new metabolites reported from marine fungi (only one before 2014 vs. 11 from 2015 to 2019) (**1**–**11**, Figure 6) [7,8].

From the deep-sea-sediment-derived fungus *Aspergillus versicolor* SD-330, two new butyrolactone-type monoterpenoids, pestalotiolactones C and D (**1** and **2**), along with a known analog, were isolated, among which the absolute configuration of the known pestalotiolactone A was determined for the first time [10]. Another deep-sea fungus, *Penicillium* sp. (YPGA11), produced two new monoterpenoids, penicipenes A and B (**3** and **4**) [11]. The marine sediment-derived fungus *Eutypella scoparia* FS46 yielded two new natural monoterpenes, eutypellol B (**5**), a rare 7-methyl oxidized 2-carene derivative, and 2-(2-hydroxy-4-methylcyclohex-3-enyl) propanoic acid (**6**, synthesized before), and the analog 2,9-epoxy-p-menth-6-en-9-ol [12]. Two new monoterpenoid *α*-pyrones, nectriapyrones C and D (**7** and **8**), and the known *α*-pyrone (nectriapyrone), were obtained from the fungus *Nectria* sp. HLS206 associated with the marine sponge *Gelliodes carnosa* [13]. The chemical investigation of a fermentation culture of the marine brown alga-endophytic strain (cf44-2) of *Trichoderma asperellum* led to the isolation of two new natural monoterpenes, (7*S*) and (7*R*)-1-hydroxy-3-*p*-menthen-9-oic acids (**9** and **10**) [14]. Moreover, using geraniol as a substrate, the biotransformation-culture broth of the marine mudflat-derived fungus *Thielavia hyalocarpa* yielded a new monoterpene glycoside, 1-*O*-(*α*-d-mannopyranosyl) geraniol (11) [15]. Unfortunately, most of these monoterpenes lack bioactivities, except for **1** and **2**, which show weak antibacterial effects (*Aeromonas hydrophilia*, *Vibrio anguillarum* for **1**, MIC = 16 and 32 μg/mL; *Vibrio harveyi*, *Edwardsiella tarda* for **2**, MIC = 32 and 32 μg/mL).

### 3.2. Sesquiterpenes

**Figure 7:**
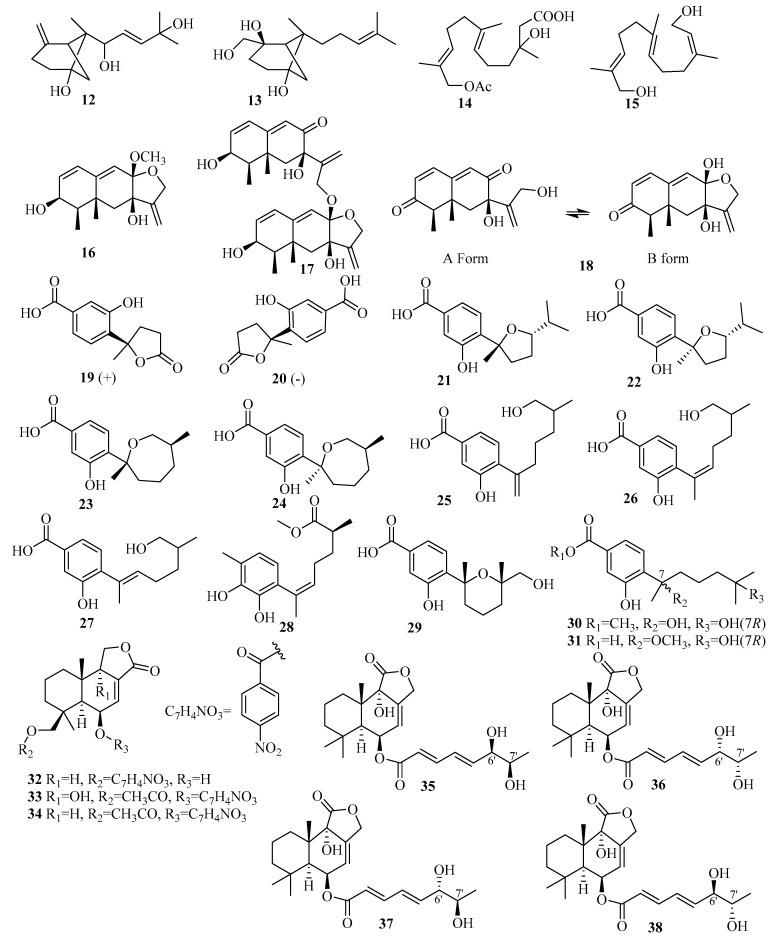
Chemical structures of sesquiterpenes (**12**–**38** from *Aspergillus* sp.).

**Figure 8:**
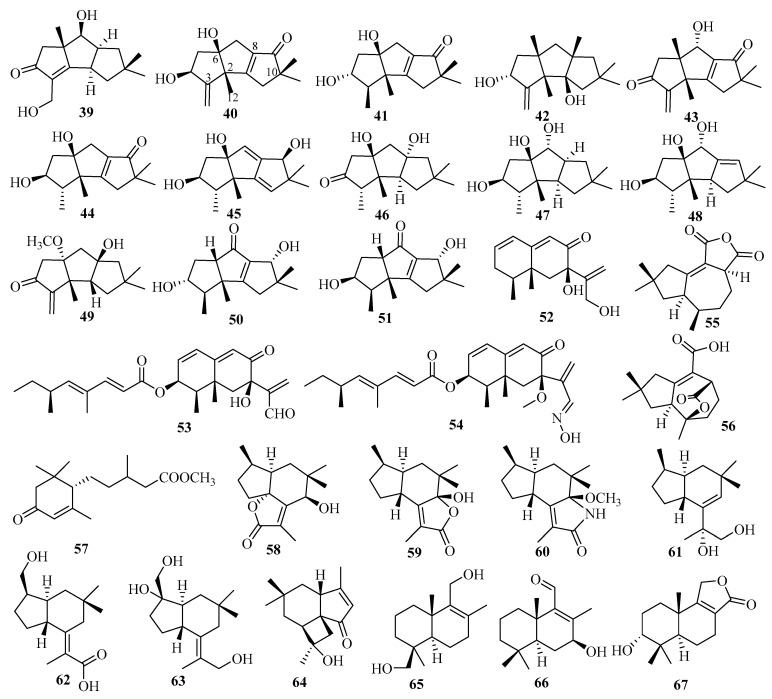
Chemical structures of sesquiterpenes (**39**–**51** from *Chondrostereum* sp., **52**–**54** from *Cochliobolus* sp., **55**–**56** from *Coriolopsis* sp., and **57**–**67** from *Diaporthe* sp.).

**Figure 9:**
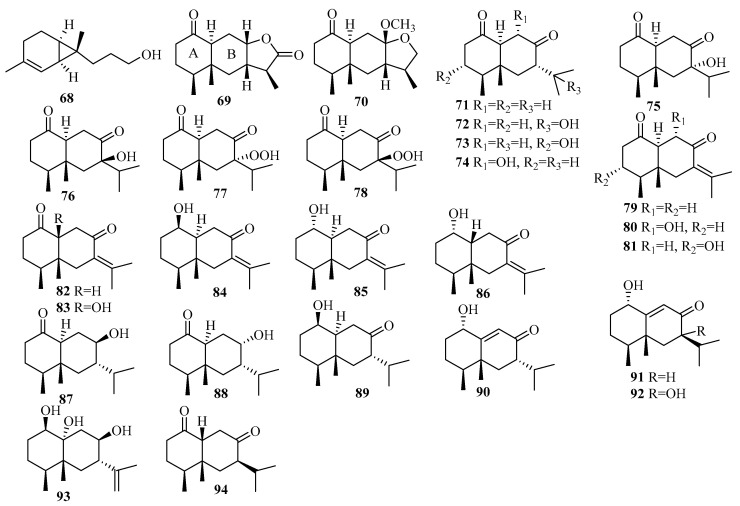
Chemical structures of sesquiterpenes (**68**–**94** from *Eutypella* sp.).

**Figure 10:**
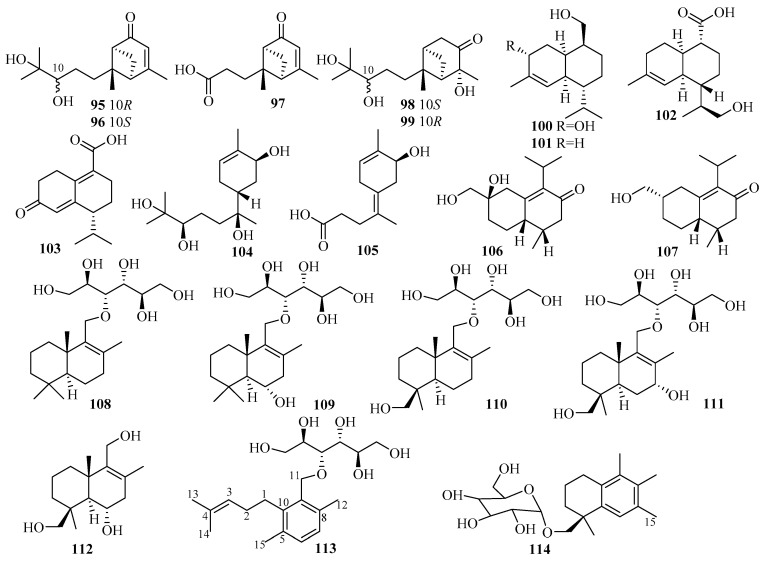
Chemical structures of sesquiterpenes (**95**–**105** from *Graphostroma* sp., **106**–**107** from *Leptosphaerulina* sp., and **108**–**114** from *Paraconiothyrium* sp.).

**Figure 11:**
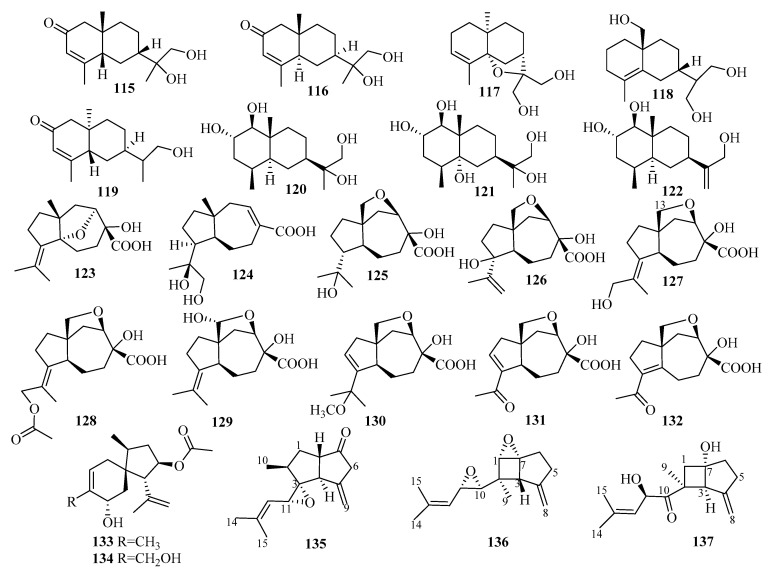
Chemical structures of sesquiterpenes (**115**–**134** from *Penicillium* sp., **135**–**137** from *Pseudallescheria* sp.).

**Figure 12:**
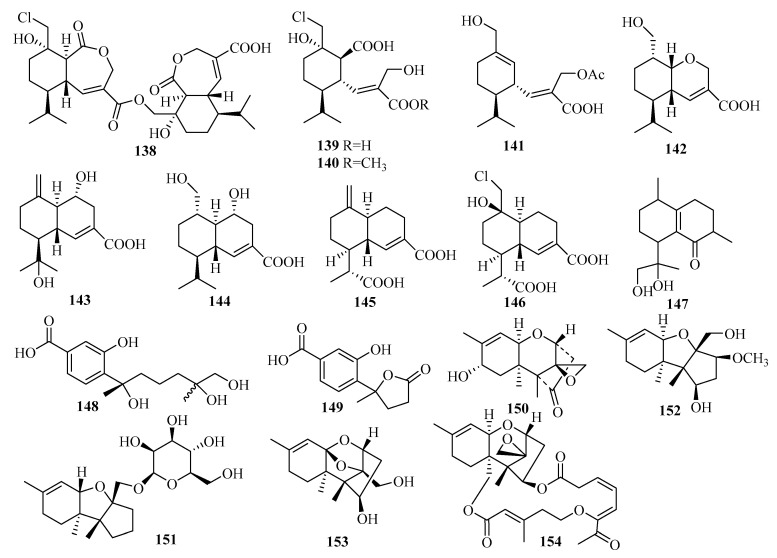
Chemical structures of sesquiterpenes (**138**–**147** from *Rhinocladiella* sp., **148**–**149** from *Scopulariopsis* sp., and **150**–**154** from *Stachybotrys* sp.).

**Figure 13:**
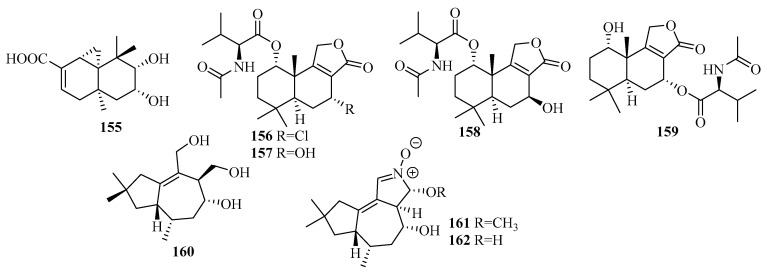
Chemical structures of sesquiterpenes (**155**–**159** from *Talaromyces* sp. and **160**–**162** from *Tinctoporellus* sp.).

**Figure 14:**
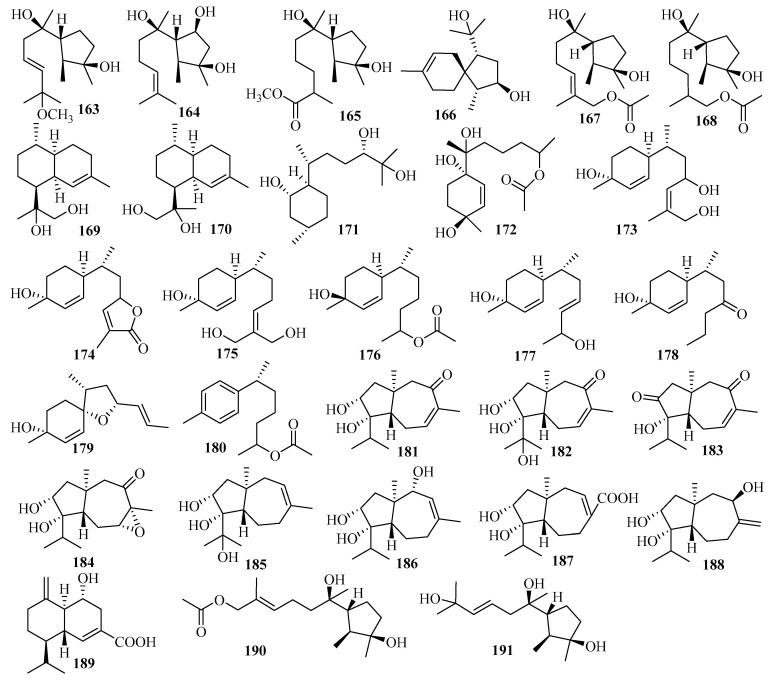
Chemical structures of sesquiterpenes (**163**–**189** from *Trichoderma* sp. and **190**–**191** from *Trichothecium* sp.).

**Figure 15:**
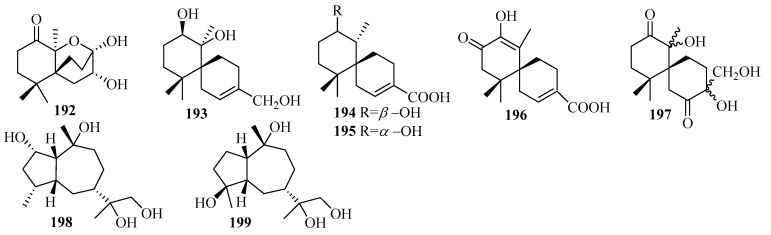
Chemical structures of sesquiterpenes (**192**–**199** from an unidentified fungus).

Sesquiterpenes are the largest group and an excellent source of terpenoids. A total of 45 research papers in 2015–2019 describe 188 new sesquiterpenes (**12**–**199**, Figure 7, Figure 8, Figure 9, Figure 10, Figure 11, Figure 12, Figure 13, Figure 14 and Figure 15) from approximately 19 genera of marine fungi, showing the significant increase in the rate of sesquiterpenes being reported based on the fact that about 208 sesquiterpenes (**111** in 2011–2014 and **97** before 2010) were reported from marine fungi by the end of 2014 [7,8].

The majority of the fungi yielding these new sesquiterpenes were mainly isolated from marine algae (44, 23%); mangrove plants (43, 23%); deep-sea sediments (38, 20%); marine animals, including sponges, coral, and the crinoid (26, 14%); and other marine sediments (23, 12%). In terms of fungal genera, the four genera of *Trichoderma* (28, 15%), *Aspergillus* (27, 14%), *Eutypella* (27, 14%), and *Penicillium* (20, 11%) are the major objects of focus for researchers in this field, while other groups of fungi, including *Chondrostereum*, *Graphostroma*, *Diaporthe*, *Rhinocladiella*, *Paraconiothyrium*, *Talaromyces*, *Stachybotrys*, *Tinctoporellus, Pseudallescheria*, *Cochliobolus*, *Trichothecium*, *Leptosphaerulina*, and *Coriolopsis*, are other significant sources of marine sesquiterpenes. Regarding biological activities, approximately 30% of the new sesquiterpenes (84 active in 281 tests of sesquiterpenes) have notable abilities, primarily cytotoxicity (30%), anti-inflammatory activity (24%), lethal toxicity (20%), antibacterial activity (15%), and enzyme inhibitor activities (8%). 

#### 3.2.1. *Aspergillus* sp.

The production of two new *β*-bergamotane sesquiterpenoids that are likely important intermediates in the biosynthesis of fumagillin and its derivatives, *E*-*β*-trans-5,8,11-trihydroxybergamot-9-ene (**12**) and *β*-trans-2*β*,5,15-trihydroxy-bergamot-10-ene (**13**), were obtained from the marine-derived fungus *Aspergillus fumigatus* YK-7 isolated from sea mud of the intertidal zone [16]. Compound **12** exhibited weak activities against the U937 cell line with IC_50_ values of 84.9 µM [16].

An investigation of the extracts from the deep-sea-sediment fungus *Aspergillus terreus* YPGA10, collected in the Yap Trench at a depth of 4159 m, furnished two new farnesol derivatives, sesquiterpenes aspterric A (**14**) and aspterric B (**15**), which are considered to be intermediates in the derivation of aspterric acid [17].

Chemical examination of the deep-sea-sediment-derived *Aspergillus* sp. SCSIOW2, treated with a combination of 1 mM suberohydroxamic acid (SBHA), a competitive histone deacetylase (HDAC) inhibitor, and 1 mM 5-azacytidine (5-AZA), a DNA methyltransferase (DNMT) inhibitor, led to the isolation of three novel eremophilane-type sesquiterpenes, dihydrobipolaroxin B–D (**16**–**18**). However, **16** and **17** might be artificial products since they could be obtained from the spontaneous intracellular acetalization reaction of dihydrobipolaroxin in water. Moreover, **18** was found to be a mixture of two equilibrium structures in a solution formed through the same acetalization reaction [18]. All of these molecules exerted moderate nitric oxide (NO)-inhibitory activities that were stimulated by lipopolysaccharides (LPSs) and interferon (IFN)-*γ* in a dose-dependent manner without any cytotoxic effects [18]. 

A pair of new norbisabolane enantiomers, named (+)-1-hydroxyboivinianic acid (**19**) and (−)-1-hydroxyboivinianic acid (**20**), were obtained from fermenting *Aspergillus versicolor* SYSU-SKS025 purified from the branches of the mangrove plant *Excoecaria agallocha* [19]. In bioactivity assays, **19** and **20** showed greater *α*-glucosidase inhibitory activity with IC_50_ values of 120.3 and 113.3 μM, respectively, compared to the IC_50_ = 350 μM of acarbose as the positive control (p.c.) [19].

An endophytic fungus *Aspergillus* sp. xy02 associated with the Thai mangrove *Xylocarpus moluccensis* yielded seven new phenolic bisabolane sesquiterpenoids: (7*R*,10*S*)-7,10-epoxysydonic acid (**21**), (7*S*,10*S*)-7,10-epoxysydonic acid (**22**), (7*R*,11*S*)-7,12-epoxysydonic acid (**23**), (7*S*,11*S*)-7,12-epoxysydonic acid (**24**), 7-deoxy-7,14-didehydro-12-hydroxysydonic acid (**25**), (*Z*)-7-deoxy-7,8-dide-hydro-12-hydroxysydonic acid (**26**), and (*E*)-7deoxy-7,8-didehydro-12-hydroxy-sydonic acid (**27**) [20]. Biologically, compounds **22**–**23**, **25**, and **27** displayed mild antibacterial activity against *Staphylococcus aureus* ATCC 25923, with IC_50_ values ranging from 31.5 to 41.9 μM [20].

The extraction of the seawater-associated fungus *Aspergillus sydowii* SW9 resulted in the isolation and identification of a novel aromatic bisabolene-type sesquiterpenoid, methyl (*R*,*E*)-6-(2,3-dihydroxy-4-methylpenyl)-2-methylhept-5-enoate (**28**) [21]. The bioassay results for **28** demonstrated selective inhibition against *Escherichia coli*, *S. aureus*, *S. epidermidis,* and *S. pneumoniae*, with MIC values ranging from 2.0 to 16 µg/mL (chloramphenicol as the p.c. with MIC values ranging from 1.0 to 2.0 µg/mL) [21].

Studies of the deep-sea-sediment-derived fungus *Aspergillus versicolor* SD-330 from the South China Sea afforded three undescribed sesquiterpenes, including one aromatic bisabolene-type, 12-hydroxysydowic acid (**29**) [22], and two bisabolane-type, ent-aspergoterpenin C (**30**) and 7-*O*-methyl-hydroxy-sydonic acid (**31**) [10]. Notably, compound **29** exhibited selective inhibitory activities against four zoonotic pathogenic bacteria, *Aeromonas hydrophilia*, *E. coli*, *Edwardsiella tarda*, and *Vibrio harveyi*, with MIC values ranging from 4.0 to 8.0 µg/mL [22]. At the same time, **30** and **31** exerted selective inhibition against *E. coli*, *E. tarda*, *V. parahaemolyticus*, and *V. harveyi*, with MIC values ranging from 2.0 to 8.0 µg/mL, and the p.c. chloramphenicol had MIC values ranging from 1.0 to 4.0 µg/mL [10].

Three relatively rare nitrobenzoyl sesquiterpenoids in nature, including two new compounds insulicolide B (**32**) and insulicolide C (**34**), and one novel natural product, 14-*O*-acetylinsulicolide A (**33**), were isolated from *Aspergillus ochraceus* Jcma1F17, which is related to the marine alga *Coelarthrum* sp. [23]. Among them, 14-*O*-acetylinsulicolide A (**33**) displayed potent inhibitory activities against three renal human tumor cell lines (HTCLs) (ACHN, OS-RC-2, and 786-O), with IC_50_ values of 4.1, 5.3, and 2.3 μM (IC_50_= 3.4, 7.0, and 4.9 μM for the p.c. sorafenib), respectively [23]. Further studies indicated that compound **33** arrested the cell cycle in the G0/G1 phase at a concentration of 1 μM and induced late apoptosis at a concentration of 2 μM after treatment of 786-O cells for 72 h [23].

Asperienes A–D (**35**–**38**), two pairs of C-6/C-7 epimeric drimane sesquiterpene esters, were successfully isolated for the first time from the marine sediment fungus *Aspergillus flavus* CF13-11. All displayed significant cytotoxic activities against HeLa, MCF-7, MGC-803, and A549 cell lines, with the IC_50_ values ranging from 1.4 to 8.3 µM (with cisplatin as p.c.) [24]. Interestingly, due to the configuration at carbon C-6′, compounds **35** and **38** had lower toxicity than **36** and **37** (with different IC_50_ values of 78, 83, 6.2, and 4.9 µM, respectively) regarding normal GES-1 cells, indicating greater potential as future antitumor agents [24].

#### 3.2.2. *Chondrostereum* sp.

The fermentation of fungus *Chondrostereum* sp., separated from the soft coral *Sarcophyton tortuosum*, led to the isolation of three new triquinane-type sesquiterpenoids: chondrosterins K–M (**39**–**41**) [25]. Chondrosterin K (**39**) is a rare hirsutane sesquiterpenoid in which a methyl group has migrated from C-2 to C-6 with a double bond between C-2 and C-3 [25]. Biological assays revealed that compounds **39**–**41** exhibited significant cytotoxicity against seven HTCLs (CNE1, CNE2, HONE1, SUNE1, A549, GLC82, and HL7702), with IC_50_ values ranging from 12.03 to 58.83 µM in vitro, with hirsutanol A used as a positive control [25]. 

Chondroterpenes A–H (**42**–**49**), possessing a 5,5,5-tricyclic hirsutane-type sesquiterpene scaffold, were isolated from the marine fungus *Chondrostereum* sp. NTOU4196, which originated from the marine red alga, *Pterocladiella capillacea* [26]. Biologically, compounds **42**, **43,** and **49** exhibited significant inhibitory effect on LPS-induced NO production in BV-2 microglial cells at a concentration of 20 μM [26]. However **49** displayed potent cytotoxic effects on BV-2 cells at a concentration of 20 μM, which implied that cell death predominantly caused decreasing NO production [26].

The soft coral-derived (*Sarcophyton tortuosum*) fungus *Chondrostereum* sp. produced two unreported hirsutane-type sesquiterpenoids, chondrosterins N and O (**50** and **51**) [27]. Compounds **50** and **51** were apparently inactive (IC_50_ values > 100 μM) for seven HTCLs [27].

#### 3.2.3. *Cochliobolus* sp.

Secondary metabolites from the marine alga-related fungus *Cochliobolus lunatus* SCSIO41401 contained three new eremophilane sesquiterpenes, dendryphiellins H–J (**52**–**54**), among which dendryphiellin J (**54**) is a rare naturally occurring aldoxime analog [28]. In bioassays, compound **53** exerted cytotoxicity against five HTCLs (ACHN, 786-O, OS-RC-2, HepG2, and SGC7901), with IC_50_ values of 1.4–4.3 μM, and antibacterial activities for three bacterial species, *S. aureus* subsp. *aureus* Rosenbach, *Erysipelothrix rhusiopathiae*, and *Pasteurella multocida* subsp. *multocida* (MIC ranging from 1.5 to 13 μg/mL), respectively [28]. Compound **54** showed cytotoxicity against HepG2 cells (IC_50_ = 5.9 μM) and ACHN cells (IC_50_ = 3.1 μM) by inducing apoptosis in a dose and time-dependent manner [28].

#### 3.2.4. *Coriolopsis* sp.

Two new tremulane sesquiterpenes, coriolopsin A (**55**) and coriolopsin B (**56**), were obtained from the EtOAc fraction of the mangrove *Ceriops tagal* endophytic fungus *Coriolopsis* sp. J5 [29]. None of these showed obvious cytotoxic or antibacterial activities in bioassays, in which three HTCLs and six strains of bacteria were tested [29].

#### 3.2.5. *Diaporthe* sp.

Further work on the mangrove plant *Rhizophora stylosa* endophytic fungus *Diaporthe* sp. SCSIO 41011 led to the isolation of one novel sesquiterpenoid, 1-methoxypestabacillin B (**57**) [30].

The chemical investigation of *Diaporthe* sp., an endophytic fungus associated with the leaves of *Rhizophora stylosa* collected in Hainan Province, yielded ten new sesquiterpenoids, including six brasilane-type, diaporols J–O (**58**–**63**); one 3,6-cycloprecapnellane compound, diaporol P (**64**); and three drimane ones, diaporols Q–S (**65**–**67**) [31]. Among them, compound **66** showed moderate cytotoxicity against the SW480 cell lines with an IC_50_ value of 8.72 μM [31].

#### 3.2.6. *Eutypella* sp.

Eutypellol A (**68**), the first norsesquiterpenoid of the sequicarene family found in nature, was obtained in the marine sediment-derived fungus *Eutypella scoparia* FS46, which was collected in the South China Sea at a depth of 292 m [12].

The chemical epigenetic manipulation of *Eutypella* sp. MCCC 3A00281, a deep-sea-sediment-associated fungus obtained from the South Atlantic Ocean at a depth of 5610 m, resulted in a significant change in the metabolite profile, including the isolation of an array of 26 eremophilane-type sesquiterpenoids, eutyperemophilanes A–Z (**69**–**94**), when treated with SBHA and a histone deacetylase inhibitor (HDI) [32]. Remarkably, most of analogs featured a *trans* fusion of rings A and B, which is uncommon for the eremophilane family in nature [32]. In terms of biological evaluation, compounds **77** and **78** significantly inhibited LPS-activated NO production in RAW264.7 macrophage cells with IC_50_ values of 8.6 and 13 μM, respectively [32].

#### 3.2.7. *Graphostroma* sp.

*Graphostroma* sp. MCCC 3A00421, a deep-sea-derived fungus obtained from the hydrothermal sulfide deposits of the Atlantic Ocean, produced two structurally corrected sesquiterpenes, (10*R*)-xylariterpenoid B (**95**) and (10*S*)-xylariterpenoid A (**96**); and nine new sesquiterpenes, xylariterpenoid E–G (**97**–**99**), khusinol B–E (**100**–**103**), and graphostromabisabol A–B (**104**–**105**) [33]. Among them, xylariterpenoid E (**97**) and graphostromabisabol B (**105**) were the first examples of trinor-bergamotane and trinor-bisabolane found in nature [33]. Interestingly, compound **100** was shown to be a potent anti-inflammatory and weak antiallergic agent, exerting an anti-inflammatory effect with an IC_50_ value of 17 μM, which is more potent than that of the p.c. aminoguanidine (IC_50_ = 23 μM), and antiallergic activity with an IC_50_ value of 150 μM, while the IC_50_ of the p.c. loratadine was 92 μM [33]. 

#### 3.2.8. *Leptosphaerulina* sp.

An investigation of extracts from the crinoid-derived fungus *Leptosphaerulina chartarum* sp. 3608 afforded two new sesquiterpenes, leptoterpenes A (**106**) and B (**107**) [34].

#### 3.2.9. *Paraconiothyrium* sp.

Seven new drimane-type sesquiterpenoids, sporulositols A–D (**108**–**111**), 6-hydro-xydiaporol (**112**), seco-sporulositol (**113**), and sporuloside (**114**), were later found in the sea mud-derived fungus *Paraconiothyrium sporulosum* YK-03 collected from the intertidal zone of Bohai Bay in Liaoning Province [35]. Structurally, compounds **108**–**111** and **113** represent the first five examples of a unique class of drimanic mannitol derivatives, while compounds **113** and **114** might belong to two new series of natural drimanes possessing an aromatic ring with a rare 4,5-secodrimanic skeleton and an unusual CH_3_-15 rearranged drimanic *α*-d-glucopyranside, respectively [35]. Compounds **108**–**114** did not show any detectable cytotoxicity against the two tested cell lines A549 and MCF-7 [35].

#### 3.2.10. *Penicillium* sp.

*Penicillium thomii* KMM 4667, isolated from the rhizome surface of *Zostera marina* (Sea of Japan), was the source of five unknown eudesmane-type sesquiterpenes, thomimarines A–E (**115**–**119**) [36,37]. Compounds **115**–**116** and **118**–**119**, at 10.0 µM, induced a significant downregulation of NO production in LPS-stimulated murine macrophages (RAW 264.7) by 24.9%, 43.4%, 20.9%, and 22.5%, respectively [36,37].

Three previously undescribed eudesmane-type sesquiterpenoids (penicieudesmols E–G (**120**–**122**)) were extracted from the mangrove plant *Ceriops tagal* endophytic fungus *Penicillium* sp. J-54 [38]. The bioactivity results showed that compound **121** has *α*-glucosidase inhibitory activity with an IC_50_ value of 2.27 mM (using acarbose as the p.c., with an IC_50_ value of 1.67 mM) [38].

Systematic isolation of the deep-sea-sediment-derived (−1420 m) fungus *Penicillium griseofulvum* from the Indian Ocean was also conducted, which resulted in the extraction of four new carotane sesquiterpenoids, penigrisacids A–D (**123**–**126**) [39]. Biologically, compound **126** exhibited weak cytotoxicity against ECA-109 tumor cells (IC_50_ = 28.7 µM) [39].

Studies of the marine-subaqueous-soil-derived fungus *Penicillium piltunense* KMM 4668, collected from Piltun Bay, Sea of Okhotsk, afforded six undescribed carotane sesquiterpenoids, piltunines A–F (**127**–**132**), among which piltunine A (**127**) was reported for the first time to be a natural product. Piltunine C (**129**) was established as a 13-hydroxy derivative of aspterric acid, and compounds **131**–**132** included 9-en-11-one and 6-en-11-one enone chromophoresin molecular structures [40]. The biological assay results showed that compound **131** induced a significant downregulation of reactive oxygen species (ROS) production (with LPS as a p.c.) at a concentration of 10 µM [40].

A chemical investigation was applied to the marine sponge-derived fungus *Penicillium adametzioides* AS-53, leading to the isolation of two unreported acorane sesquiterpenes, adametacorenols A (**133**) and B (**134**) [41]. As shown by cytotoxicity assay, compound **134** exerted selective activity against the NCI-H446 cell line (IC_50_ = 5.0 μM) [41].

#### 3.2.11. *Pseudallescheria* sp.

Pseudapene A (**135**), with a unique 2-methyl-5-methylene-3-(2-methyl)-dicyclo(3, 3, 0)-octane carbon skeleton, and pseudapenes B–C (**136**–**137**), possessing an unprecedented 2-methyl-4-methylene-2-(2-methylpent-2-ene)-dicyclo(3,2,0)-heptane chemical scaffold, were isolated from the marine-derived fungus *Pseudallescheria apiosperma* F52-1 [42]. 

#### 3.2.12. *Rhinocladiella* sp.

The mangrove-derived fungus *Rhinocladiella similis* from the fresh leaves of *Acrostichum aureum* (Pteridaceae) was the source of ten new sesquiterpenoid derivatives, rhinomilisins A–J (**138**–**147**), corresponding to one dimeric sesquiterpenoid, four new heptelidic acid derivatives, and five new cadalene-type derivatives [43]. The cytotoxicities of all these isolated compounds were evaluated against the mouse lymphoma cell line L5178Y; compounds **138** and **144** exhibited moderate activity with IC_50_ values of 5.0 and 8.7 μM, respectively [43].

#### 3.2.13. *Scopulariopsis* sp.

Two new phenolic bisabolane-type sesquiterpenes, 11,12-dihydroxysydonic acid (**148**) and 1-hydroxyboivinianic acid (**149**), were later found in solid rice cultures of the marine-derived fungus *Scopulariopsis* sp., which was obtained from the fresh crushed inner tissues of the Red Sea hard coral *Stylophora* sp. near the coastline of the Ain El-Sokhna area, Red Sea, Egypt [44].

#### 3.2.14. *Stachybotrys* sp.

A new trichothecene-type sesquiterpenoid, stachybotrichodermone A (**150**), was obtained from the cultures of the sponge-derived fungus *Stachybotrys* sp. HH1 ZDDS1F1-2 [45]. A bioassay-guided investigation of the fermentation of the sponge (*Niphates recondite*)-associated fungus *Stachybotrys chartarum* WGC-25C-6, collected from the inner coral reef in Beibuwan Bay, resulted in the isolation and identification of four trichothecene-based sesquiterpenes, chartarenes A–D (**151**–**154**) [46]. Notably, trichothecene (with a sugar linkage like **151**) was found in nature for the first time [46]. Compounds **151**–**154** exerted potent or selective inhibition against a panel of HTCLs, including HCT-116, HepG2, BGC-823, NCI-H1650, and A2780 [46]. Specifically, at a concentration of less than 10 μM, compounds **151**–**154** showed inhibition against HCT-116 with IC_50_ values ranging from 0.74 to 5.58 μM, while **151**, **153**, and **154** exerted inhibitory effects against HepG2 (IC_50_ = 0.90–3.95 μM) and A2780 (IC_50_ = 0.69–2.38 μM) [46]. Moreover, **151** and **154** exhibited inhibitory activities against BGC-823 with IC_50_ values of 2.87 and 0.68 μM, respectively, while **153** and **154** inhibited NCI-H1650 (IC_50_ = 2.58 and 1.23 μM, respectively) [46]. Additionally, these compounds presented potent inhibition against the tumor growth-related tyrosine multiple kinase targets FGFR3, IGF1R, PDGFRb, and TRKB at a concentration of <10.4 μM, except for **151,** which showed weak activity, with IC_50_ > 25 μM [46]. 

#### 3.2.15. *Talaromyces* sp.

An investigation of the extracts from a marine mud-related fungus *Talaromyces purpurogenus* PP-414 collected on a coastal beach furnished the first thujopsene-type sesquiterpenoid, 9,10-diolhinokiic acid (**155**), possessing a 9,10-diol moiety [47]. Compound **155** exhibited moderate antiproliferative activities against two HTCLs (A549 and HL-60), with IC_50_ values of 12.6 and 35.7 µM, respectively [47].

The extraction of *Talaromyces minioluteus* (*Penicillium minioluteum*) PILE 14-5, a marine sponge-derived fungus (collected in Pilae Bay, Phi Phi Island, Thailand), led to the production of four new sesquiterpene lactones (**156**–**159**), namely, minioluteumides A–D, which are sesquiterpene lactones conjugated with *N*-acetyl-l-valine that rarely occur in nature [48]. The result of the cytotoxic activity assay of the HepG2 cancer cell lines revealed that compounds **156** and **159** exerted cytotoxicity, with IC_50_ values of 50.6 and 57.0 µM, respectively [48]. 

#### 3.2.16. *Tinctoporellus* sp.

Three new tremulene terpenes, **160**–**162**, two of which bear structurally unprecedented 2-hydroxy or 2-methoxy-3,4-dihydro-2*H*-pyrrole *N*-oxide moieties, were obtained from the RBBR degradation medium as the products of the metabolism of *Tinctoporellus* sp. CBMAI 1061, which was isolated from a specimen of the sponge *Dragmacidon reticulatum* [49]. 

#### 3.2.17. *Trichoderma* sp.

Secondary metabolites from *Trichoderma harzianum* X-5, an endophytic fungus associated with the marine brown alga *Laminaria japonica* from the Chang Islands, included three cyclonerane sesquiterpenes, 11-methoxy-9-cycloneren-3,7-diol (**163**), 10-cycloneren-3,5,7-triol (**164**), and methyl-3,7-dihydroxy-15-cycloneranate (**165**) in addition to one acorane sesquiterpene, 8-acoren-3,11-diol (**166**) [50]. In the biological evaluation, they all exhibited growth inhibition of four phytoplankton species (*Chattonella marina*, *Heterosigma akashiwo*, *Karlodinium veneficum,* and *Prorocentrum donghaiense*), among which **163** was most active against *C. marina* and *K. veneficum* with IC_50_ values of 0.66 and 2.2 μg/mL, respectively [50].

Two undescribed cyclonerane sesquiterpenoid derivatives, (10*E*)-12-acetoxy10-cycloneren-3,7-diol (**167**) and 12-acetoxycycloneran-3,7-diol (**168**), were isolated from the cultures of the marine sediment-derived fungus *Trichoderma harzianum* P1-4 collected in the Bohai Sea [51]. 

Further work on an endophytic fungal strain from the marine red alga *Gracilaria verrucose* collected from the Yangma Island, *Trichoderma asperellum* A-YMD-9-2, led to the production of two new sesquiterpenoids, 4-cadinen-11,12-diol (**169**) and 4-cadinen-11,13-diol (**170**) [52]. During the bioactivity assay, compounds **169**–**170** showed potent inhibition of four marine phytoplankton species related to red tides: *Chattonella marina*, *Heterosigma akashiwo*, *Karlodinium veneficum*, and *Prorocentrum donghaiense,* with IC_50_ values ranging from 1.1 to 8.9 μg/mL and weak inhibition against marine pathogenic bacteria, including four different strains of *Vibrio* and a strain of *Pseudoalteromonas citrea,* at 40 μg/disk [52]. 

Research on secondary metabolites of the strain *Trichoderma asperellum* cf44-2, which is related to the marine brown alga *Sargassum* sp. collected from the Zhoushan Islands, led to a new bisabolane sesquiterpene, bisabolan-1,10,11-triol (**171**), and a novel norbisabolane sesquiterpene, 12-nor-11-aceto-xybisabolen-3,6,7-triol (**172**) [14]. The evaluation of compounds **171**–**172** for the inhibition of four marine phytoplankton species and four marine-derived pathogenic bacteria (the same test strain used for compounds **169**–**170**) revealed that they all resulted in growth inhibition of the four phytoplankton species tested and weak antibacterial activities were observed against the four tested bacteria, with inhibitory zone diameters of 6.3–7.5 mm at 20 µg/disk [14]. 

From the culture of *Trichoderma asperellum* Y6–2, originating from the surface of the marine red alga *Chondrus ocellatus*, eight new bisabolane derivatives, trichobisabolins A–H (**173**–**180**), were obtained [53]. All displayed inhibition against four phytoplankton species (*H. akashiwo*, *P. donghaiense*, *C. marina,* and *K. veneficum*), among which **180**, with IC_50_ values ranging from 1.9 to 3.8 μg/mL, was more active than the others [53]. In addition, **176** and **180** showed weak lethality to the marine zooplankton *Artemia salina*, with IC_50_ values of 48 and 62 μg/mL, respectively [53]. 

Further research on the marine red alga-associated fungus *Trichoderma virens* Y13-3 yielded two types of sesquiterpenes: eight new carotene sesquiterpenes, trichocarotins A–H (**181**–**188**), and a new cadinene sesquiterpene, trichocadinin A (**189**) [54]. Compounds **183**–**185** and **188** showed potent inhibition against the four phytoplankton species (*H. akashiwo*, *P. donghaiense*, *C. marina,* and *K. veneficum*), (IC_50_ = 0.24–12 μg/mL), especially against *C. marina*, with IC_50_ values ranging from 0.24 to 1.2 μg/mL, which indicates that they might be promising agents to control harmful algal bloom [54]. 

#### 3.2.18. *Trichothecium* sp.

A culture of the marine-derived fungus *Trichothecium roseum* from marine driftwood, collected from the intertidal zone of Lingshan Island, was the source of two cyclonerodiol sesquiterpenes: cyclonerodiol C–D (**190**–**191**) [55]. The biological evaluation showed that **191** produces moderate antifungal activity against *Valsa mali*, with a MIC value of 64 µg/mL; meanwhile, **190** exhibited weak bioactivity against *V. mali* and *rhellozoctonia cerealis* [55].

#### 3.2.19. Unidentified Fungus

Six new chamigrane sesquiterpenes, merulinols A–F (**192**–**197**), were isolated from the culture of the endophytic fungus XG8D belonging to the family Meruliaceae and associated with the Thai mangrove *Xylocarpus granatum* [56]. Among them, merulinol A (**192**) was a nor-chamigrane with a novel tricyclic ring system, whereas compounds **193**–**197** were 6/6 spirobicyclic charmigrane sesquiterpenes [56]. Compounds **194** and **195** exhibited selective cytotoxicity toward gastric KATO-3 cells, with IC_50_ values of 35.0 and 25.3 µM, respectively [56].

Two new sesquiterpenoids, 2*α*-hydroxyxylaranol B (**198**) and 4*β*-hydroxyxylaranol B (**199**), were obtained from the fermentation of the endophytic fungus J3 isolated from the leaf of the mangrove plant *Ceriops tagal* collected from the mangrove reserve of Dong Zhai Gang [57].

### 3.3. Diterpenes

**Figure 16:**
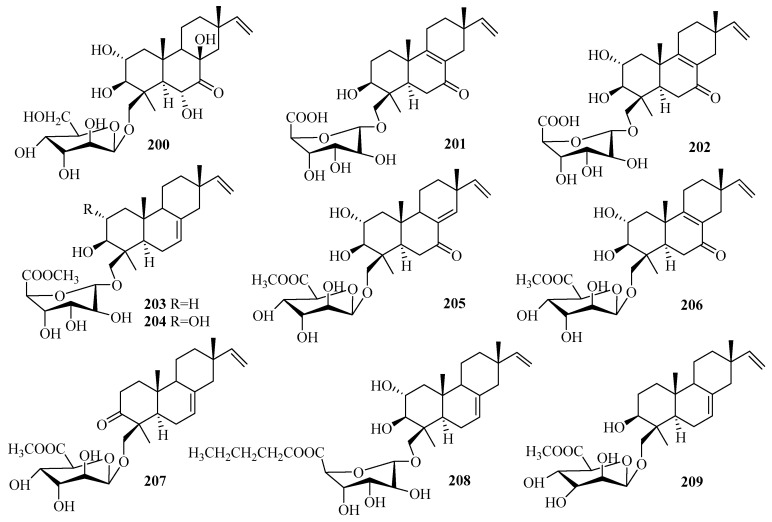
Chemical structures of diterpenes (**200**–**209** from *Acremonium* sp.).

**Figure 17:**
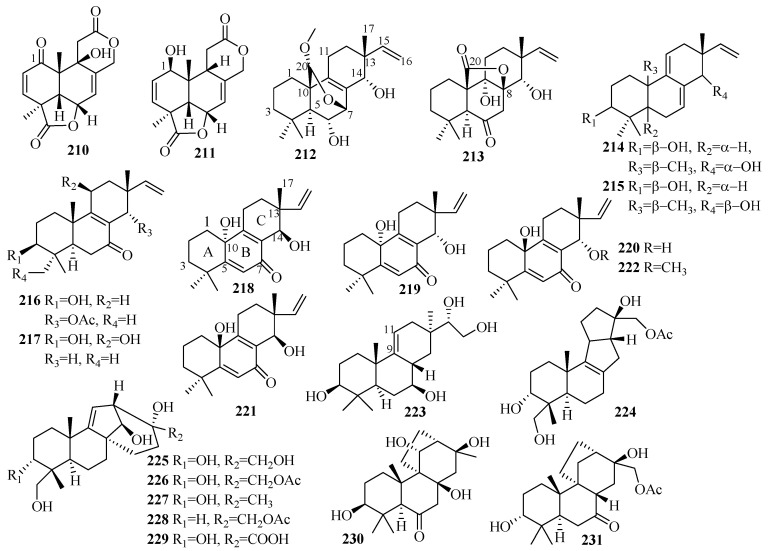
Chemical structures of diterpenes (**210**–**222** from *Aspergillus* sp. and **223**–**231** from *Botryotinia* sp.).

**Figure 18:**
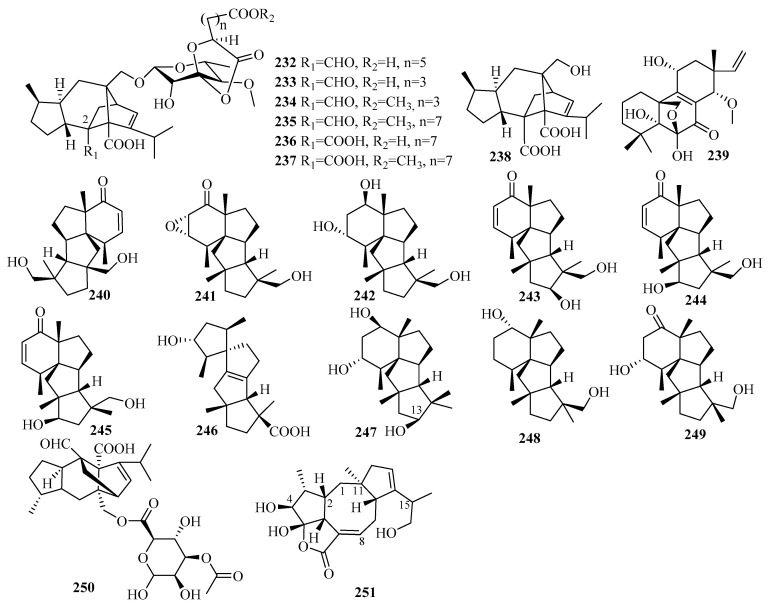
Chemical structures of diterpenes (**232**–**238** from *Curvularia* sp., **239** from *Epicoccum* sp., **240**–**250** from *Penicillium* sp., and **251** from *Talaromyces* sp.).

**Figure 19:**
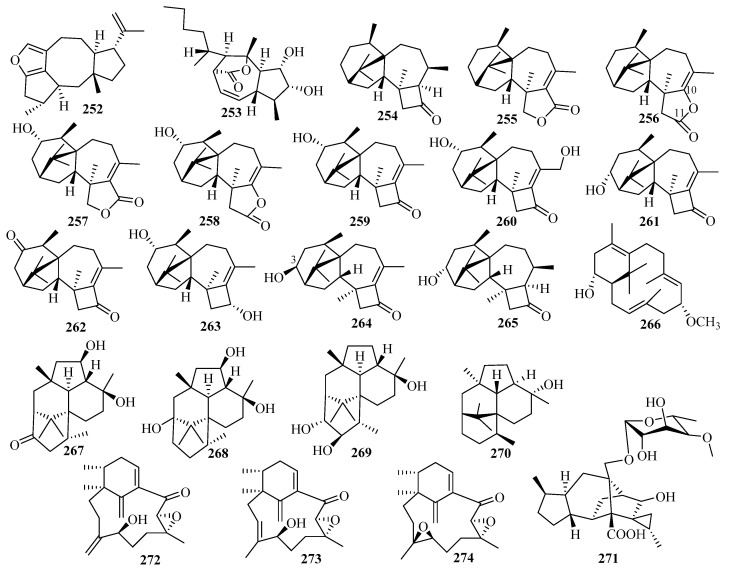
Chemical structures of sesquiterpenes (**252**–**271** from *Trichoderma* sp. and **272**–**274** from an unidentified fungus).

Diterpenes are a significant class of terpenoids with diverse structures and notable bioactivities. The current research (25 articles from 2015 to 2019) reported 75 undescribed diterpenes (**200**–**274**, Figure 16, Figure 17, Figure 18 and Figure 19) in total from diverse marine fungi compared to 77 diterpenes (42 in 2011–2014, with 35 before 2010) described in 36 papers up to 2014 [7,8], which indicates that the number of diterpenes isolated from marine fungi continues to increase.

The newly reported diterpenes were obtained from fungi associated with diverse marine habitats; the number of fungi from marine animals (28, 37%) was the highest, followed by those from deep-sea sediments (17, 23%), and then seawater (12, 16%), algae (10, 13%), other marine sediments (6, 8%), and mangrove plants (2, 3%). These marine fungi can be divided into nine groups of genera, in which five main genera each account for more than 10%: *Trichoderma* (27%), *Aspergillus* (17%), *Penicillium* (15%), *Acremonium* (13%), *Botryotinia* (12%); some groups occur in relatively small amounts; namely, *Curvularia*, *Talaromyces*, *Epicoccum*, and one unidentified fungus. The results of the biological evaluations conducted for new diterpenes show that 38% of them (40 active in 106 tested diterpenes) offer diverse inhibitory activities, most of which displayed lethal toxicity (35%), cytotoxicity (20%), and antibacterial (17.5%) and anti-inflammatory (15%) activities. 

#### 3.3.1. *Acremonium* sp.

Ten undescribed diterpene glycosides, virescenosides Z_9_–Z_18_ (**200**–**209**), were identified as secondary metabolites of the marine-derived fungus *Acremonium striatisporum* KMM 4401 associated with the holothurian *Eupentacta fraudatrix* on a wort agar medium treated with potassium bromide [58]. Structurally, compound **200** is an altroside featuring a new 7-oxo-isopimara-15-en-2*α*,3*β*,6*α*,8*β*-tetraol aglycon. **203**–**207** were determined to be monosides coupled with unique methyl esters of altruronic acid as their sugar moieties [58]. The carbohydrate chain of virescenoside Z18 (**209**) was identified as the methyl ester of mannuronic acid [58]. The bioactivity assay revealed that compounds **200**, **201**, **203**, and **204** induced a significant downregulation of ROS production in macrophages stimulated with LPS at a concentration of 10 µM, especially for virescenoside Z_10_ (**201**), thereby decreasing the ROS content in the macrophages by 45% [58]. Furthermore, compounds **201** and **204** displayed moderate downregulation of NO production in LPS-stimulated macrophages at a concentration of 1 µM [58].

#### 3.3.2. *Aspergillus* sp.

The team of Li et al. conducted a further investigation on the deep-sea-sediment-derived fungus *Aspergillus wentii* SD-310, collected in the South China Sea at a depth of 2038 m, which led to the isolation of 13 new diterpenoids, including two tetranorlabdane diterpenoids, asperolides D–E (**210**–**211**) [59], six isopimarane-type diterpenoids, wentinoids A–F (**212**–**217**) [60], four new, uncommon 20-nor-isopimarane diterpenoid epimers, aspewentins I–L (**218**–**221**), and a new methylated derivative, aspewentin M (**222**) [61]. Among them, wentinoid A–B (**212**–**213**) belonged to a rarely-described class of tetracyclic isopimaranes—specifically wentinoid A (**212**), which contains an unusual 20-acetal moiety and 7,20-oxabridged functionality, whereas wentinoid B (**213**) possesses a novel 8,20-lactone-bridged scaffold [60]. Moreover, compounds **218**–**222** are rare examples of 20-nor-isopimarane analogs coupled with a cyclohexa-2,5-dien-1-one moiety at ring B [61]. The results of the biological assay revealed that compound **211** exerts cytotoxic activities against HeLa, MCF-7, and NCI-H446 cell lines, with IC_50_ values of 10.0, 11.0, and 16.0 µM, respectively, and moderate activity against the aquatic bacteria *Edwardsiella tarda*, with a MIC value of 16 µg/mL [59]. However, compound **210** did not display this activity, possibly due to the keto substitution at C-1 [59]. Thus, compound **212** might prove useful as an antifungal agent for its selective inhibition of four plant pathogenic fungi, including *Phytophthora parasitica, Fusarium oxysporum* f. sp. *lycopersici*, *Fusarium graminearum*, and *Botryosphaeria dothidea*, with MIC values of 8.0, 4.0, 1.0, and 4.0 µg/mL, respectively, compared to the p.c., amphotericin B (MIC = 2.0, 1.0, 1.0, and 2.0 µg/mL, respectively) [60]. Furthermore, compound **218** showed activity against *E. coli* (MIC = 32 µg/mL), while compounds **218** and **219** possessed potent inhibitory activities toward three marine bacteria (*E. tarda*, *V. harveyi*, and *V. parahaemolyticus*), each with a MIC value of 8.0 µg/mL [61]. Compound **222** exhibited activity against *F. graminearum* with a MIC value of 4.0 µg/mL (the same MIC as for the p.c. amphotericin B) [61]. Moreover, compounds **218**–**219** were more active against pathogenic bacteria than **220**–**222**, suggesting that compounds with an *R* absolute configuration at C-10 are more active than those with *S* configuration [61]. Compared to **218**–**221**, compound **222** offered better inhibition of *F. graminearum* with methoxylation at C-14 [61].

#### 3.3.3. *Botryotinia* sp.

Further work was done by Niu et al. on the fermentation broth of the *Botryotinia fuckeliana* MCCC obtained from the deep seawater in the western Pacific Ocean at a depth of 5572 m [62,63]. This led to the isolation of a new pimarane diterpenoid, botryopimarene A (**223**), featuring a Δ^9(11)^ double bond, a rare moiety in the pimarane family [62], and eight unprecedented diterpenoids: botryotins A–H (**224**–**231**), representing three novel carbon skeletons with 6/6/5/5 (**224**), 6/6/5/6 (**225**–**229**), and 6/6/6/5 (**230** and **231**) tetracyclic scaffolds [63]. Compounds **224**–**231** proved inactive against six HTCLs (HL-60, BEL-7402, BIU-87, PANC-1, HeLa-S3, and ECA109) (IC_50_ > 20 μM). However, **224** exhibited moderate antiallergic activity in RBL-2H3 cells (IC_50_ = 0.2 mM) with the loratadine as p.c. (IC_50_ = 0.1 mM) [62,63].

#### 3.3.4. *Curvularia* sp.

Secondary metabolites from the marine fungus *Curvularia hawaiiensis* TA2615 (related to the zoanthid *Palythoa haddoni* collected from the Weizhou coral reefs in the South China Sea) contained a new sordaricin tetracyclic diterpene named sordaricin B (**238**) and six new sordarin tetracyclic diterpene glycosides, moriniafungins B–G (**232**–**237**)—the first instances of sordarins from marine-derived fungi each bearing a sordarose with a rare spiro 1,3-dioxolan-4-one ring [64]. Among them, compound **235** displayed strong antifungal activity against *Candida albicans* ATCC10231, with a MIC value of 2.9 μM [64]. The differences in antifungal activity among these compounds indicate that the glycosyl moiety, the length of the aliphatic acid side chain, and C-2 carboxylic acid might have impacts on antifungal activity [64].

#### 3.3.5. *Epicoccum* sp.

One unreported isopimarane diterpene (**239**) was sourced from the *Apostichopus japonicus*-associated fungus *Epicoccum* sp. HS-1 [65]. In the bioassay, **239**, with IC_50_ values of 4.6 μM, exhibited greater effective inhibition against *α*-glucosidase than the p.c. resveratrol, with IC_50_ = 31.2 μM [65]. 

#### 3.3.6. *Penicillium* sp.

A further study on the deep-sea-derived fungus *Penicillium commune* MCCC 3A00940 yielded three new cyclopiane diterpenes exhibiting a rare rigid 6/5/5/5 fused tetracyclic ring framework, conidiogenone J–K (**240**–**241**) and conidiogenol B (**242**), among which conidiogenone J (**240**) is notably the first naturally occurring cyclopiane diterpene enantiomer [66]. However, all these compounds were devoid of bioactivity when tested for antiallergic effects in immunoglobulin E (IgE)-mediated rat basophilic leukemia RBL2H3 cells [66].

An investigation of the extracts from *Penicillium* sp. TJ403-2, a sea sediment-derived fungus, yielded three new rare diterpenes identified as 13*β*-hydroxy conidiogenone C (**243**), 12*β*-hydroxy conidiogenone C (**244**), and 12*β*-hydroxy conidiogenone D (**245**) [67]. Regarding anti-inflammatory activity against LPS-induced NO production in RAW 264.7 cells, **243** displayed notable inhibitory potency, with an IC_50_ value of 2.19 μM—three-fold lower than the p.c. indomethacin (IC_50_ = 8.76 μM) [67]. Moreover, further experiments showed that **243** could significantly inhibit the production of cell cytokines (IL-1*β*, IL-13, TNF-*α*, GM-CSF, MIP-1*β*, and MCP-1), suppress inducible nitric oxide synthesis (iNOS) and cyclooxygenase-2 (COX-2) protein expression in a dose-dependent manner, and abolish the nuclear translocation of NF-*k*B p65 in LPS-activated RAW264.7 cells [67]. From these data, it is apparent that **243** is a promising starting point for the development of new anti-inflammatory agents due to its inhibition of the NF-*k*B-activated pathway [67].

The deep-sea-sediment fungus *Penicillium granulatum* MCCC 3A00475, occurring in the Prydz Bay of Antarctica at a depth of 2284 m, produced a rare novel spiro-tetracyclic diterpene, spirograterpene A (**246**), reported to be the second example of a diterpene featuring a 5/5/5/5 spirocyclic carbon skeleton [68]. Biologically, compound **246** showed modest antiallergic activity against IgE-mediated rat mast RBL-2H3 cells with 18% inhibition, compared to 35% inhibition for the p.c. loratadine at the same concentration of 20 μg/mL [68]. 

The EtOAc extract of *Penicillium* sp. YPGA11, a deep-sea-sediment fungus collected in the West Pacific Ocean at a depth of 4500 m, resulted in the isolation of three new cyclopiane diterpenes, conidiogenols C–D (**247**–**248**) and conidiogenone L (**249**) [69]. Structurally, compound **247** is the second example of cyclopianes bearing a hydroxyl group at C-13, while **248** is the third example of conidiogenols (coupled with a distinct *α*-oriented 1-hydroxy group) [69]. After the bioassay study demonstrated the inhibitory effects against five esophageal HTCLs (EC109, KYSE70, EC9706, KYSE30, and KYSE450), compounds **247** and **249** displayed weak inhibitory effects with inhibition rates less than 36% at an initial concentration of 50 μM [69]. Compound **248** exerted moderate antiproliferative effects, with IC_50_ values ranging from 36.80 to 54.7 μM (cisplatin as the p.c.) [69]. 

A new glycosyl ester identified as xylarinonericin E (**250**) was later found in the fermentation broth of the fungus *Penicillium* sp. H1 from the sediments of the Beibu Gulf [70]. Compound **250** showed moderate antifungal activity against *Fusarium oxysporum* f. sp. *cubense,* with an MIC value of 32.0 μM [70].

#### 3.3.7. *Talaromyces* sp.

Characterized as a new diterpenoid bearing a novel tetracyclic fusicoccane framework with an unexpected hydroxyl at C-4, roussoellol C (**251**) was obtained from the laboratory cultures of *Talaromyces purpurogenus* PP-414 originating from a mud sample collected on a coastal beach [47]. Additionally, compound **251** exhibited moderate antiproliferative activities against four HTCLs (SW480, HL-60, A549, and MCF-7), with IC_50_ values ranging from 6.5 to 25.8 µM [47]. Furthermore, **251** showed significant selectivity toward MCF-7 cells, with an IC_50_ value of 6.5 µM but an IC_50_ value of 10.9 µM against HL-60, contrary to the expectation that cytotoxic natural products will display greater activities against HL-60 cells due to their high sensitivity in the assay [47]. Thus, the selectivity of **251** against MCF-7 makes it a promising lead compound for further study [47].

#### 3.3.8. *Trichoderma* sp.

The ongoing studies of Liang, X. R. et al. on the fungus *Trichoderma citrinoviride* cf-27 associated with the fresh tissue of the marine brown alga *Dictyopteris prolifera* afforded the first *Trichoderma*-derived and furan-bearing fusicoccane diterpene trichocitrin (**252**) [71], and a novel norditerpene, citrinovirin (**253**), with an unprecedented skeleton [72]. The bioactivity results showed that **252** can exert antibacterial activity against *E. coli* with an inhibitory diameter of 8.0 mm at 20 μg/disc and possesses anti-microalgal capability against *Prorocentrum donghaiense* with 54.1% growth inhibition at 80 μg/mL [71]. Compound **253** inhibited the growth of *S. aureus* with an MIC value of 12.4 μg/mL, displayed toxicity against the marine zooplankton *Artemia salina* with an LC_50_ value of 65.6 μg/mL, and additionally achieved 14.1–37.2% inhibition of three marine phytoplankton species (*C. marina*, *H. akashiwo,* and *P. donghaiense*) at 100 μg/mL but promoted the growth of one marine phytoplankton *Scrippsiella trochoidea* [72]. 

The fermentation culture of the endophytic fungus *Trichoderma* sp. Xy24 from the mangrove plant *Xylocarpus granatum*, collected in the Sanya district, was the source of two new harziane diterpenoids, (9*R*, 10*R*)-dihydro-harzianone (**254**) and harzianelactone (**255**) [73]. Among them, **254** was the reductive product of harzianone, and **255** was the Baeyer–Villiger monooxygenase-catalyzed oxidation product of harzianone [73]. In terms of in vitro biological evaluation, compound **254** exerted selective cytotoxicity on the HeLa and MCF-7 cell lines with IC_50_ values of 30.1 and 30.7 µM, respectively, whereas **255** was inactive at 10 mM [73].

The cultivation of *Trichoderma longibrachiatum* A-WH-20-2, an endophyte from the marine red alga *Laurencia okamurai*, yielded one new harziane lactone with an ester linkage between C-10 and C-11, deoxytrichodermaerin (**256**), which was possibly an oxidation product of harzianone [74]. Deoxytrichodermaerin (**256**) and the other two isolates (harzianol A and harzianone) were assayed for their inhibition of four marine phytoplankton species (*C. marina*, *H. akashiwo*, *K. veneficum*, and *P. donghaiense*) with IC_50_ values ranging from 0.53 to 2.7 µg/mL [74]. The toxicity of **256** against the marine zooplankton *A. salina* was also evaluated (LC_50_ = 19 µg/mL) and revealed that the lactone unit in deoxytrichodermaerin may contribute slightly to these activities [74]. 

The chemical investigation was applied to the soft coral-derived fungus *Trichoderma harzianum* XS20090075 in the South China Sea, resulting in the production of seven secondary metabolites, including two new harziane diterpene lactones possessing a 6/5/7/5-fused carbocyclic core containing a lactone ring system, harzianelactones A and B (**257** and **258**), and five new harziane diterpenes, harzianones A–D (**259**–**262**) and harziane (**263**) [75]. Specifically, **257** and **258** represent a unique type of harziane diterpene lactone that originated from harziane diterpenes though Baeyer–Villiger monooxygenase-catalyzed oxidation [75]. In the bioactivity study, the above compounds (except for **262**) showed obvious phytotoxicity against the seedling growth of amaranth and lettuce at a concentration of 200 ppm [75]. Among them, compounds **257**, **259**, **260**, and **261** were more effective for the complete inhibition of seed germination against amaranth at 200 μg/mL and were still effective at lower concentrations (50 μg/mL) compared to the p.c. glyphosate [75]. However, none was found to inhibit the root growth of lettuce at 200 ppm [75]. In short, the tested compounds seemed to cause weaker inhibition to lettuce than to amaranth and had stronger toxicity on root growth than hypocotyl [75]. Notably, this was the first report of the phytotoxicity of harziane diterpenes from *Trichoderma* spp. [75]. 

The search for antagonistic metabolites from *Trichoderma asperellum* A-YMD-9-2, an endophytic fungal from the marine red alga *Gracilaria verrucose*, led to the isolation of one new harziane diterpenoid, 3*S*-hydroxyharzianone (**264**), which may be an intermediate in the biosynthesis of harziandione from harzianone [52]. Compound **264** could strongly inhibit a wide spectrum of red tide-related phytoplankton species (*C. marina*, *H. akashiwo*, *K. veneficum*, and *P. donghaiense*) with IC_50_ values ranging from 3.1 to 7.7 μg/mL [52]. A structure–activity relationship analysis revealed that the hydroxy group at C-3 of **264** greatly contributes to its inhibitory ability. During the antibacterial assay, **264** exhibited weak inhibition against five marine-derived pathogenic bacteria (four different strains of *Vibrio* and a *P. citrea*), at 40 μg/disc [52].

As a result of the continuing endeavor to research secondary metabolites from brown alga, the *Laminaria japonica*-derived strain *Trichoderma harzianum* X5, two new structurally related diterpenes, 3*R*-hydroxy-9*R*,10*R*-dihydroharzianone (**265**) and 11*R*-methoxy-5,9,13-proharzitrien-3-ol (**266**), were isolated and identified [50]. They were all evaluated for their ability to inhibit four marine phytoplankton species, as with the above strains tested for **264**, and exhibited growth inhibition of these tested species (IC_50_ = 1.2–70 μg/mL) [50]. Notably, compound **266** exhibited potent inhibition of *H. akashiwo* and *P. donghaiense* with IC_50_ values in the low μg/mL range [50].

Trichodermanins C–E (**267**–**269**), three undescribed diterpenes with a rare fused 6-5-6-6 ring system, were isolated from the fungus *Trichoderma harzianum* OUPS-111D-4 from a piece of a marine sponge *Halichondria okadai* [76]. Significantly, this was the first determination of the absolute configurations of **267**–**269** [76]. In a primary screen for antitumor activity, **267** exerted strong inhibition against the cell lines P388, HL-60, and L1210, with IC_50_ values ranging from 6.8 to 7.9 µM, in which the p.c. 5-fluorouracil exhibited IC_50_ values ranging from 4.5 to 6.1 µM [76].

An investigation of the extracts from the fungus *Trichoderma asperellum* d1-34 that occurs on marine brown alga afforded one unknown diterpene antipode, (+)-wickerol A (**270**) [77]. The bioactivity assay showed that compound **270** could inhibit *E. coli* and *S. aureus* with the same inhibitory diameters of 8.0 mm at 30 μg/disc, and lethal activity was displayed against *A. salina* with an LC_50_ value of 12.0 μg/mL [77].

A new sordarin derivative, trichosordarin A (**271**), bearing a unique norditerpene aglycone, was discovered from the extracts of a marine sediment-derived fungal strain collected in the Bohai Sea, *Trichoderma harzianum* R5 [78]. It was assayed to be toxic to the marine zooplankton *A. salina* with an LC_50_ value of 233 μM, but was weak in inhibiting two marine phytoplankton species (*Amphidinium carterae* and *Phaeocysti globosa*), with inhibition rates of 20.6% and 8.1%, respectively, at 100 μg/mL [78].

#### 3.3.9. Unidentified Fungus

An unidentified marine fungus associated with the marine brown alga *Ishige okamurae* (Tateishi, Kanagawa Prefecture) furnished three novel diterpenes, phomactins N–P (**272**–**274**) [79].

### 3.4. Sesterterpenes

**Figure 20:**
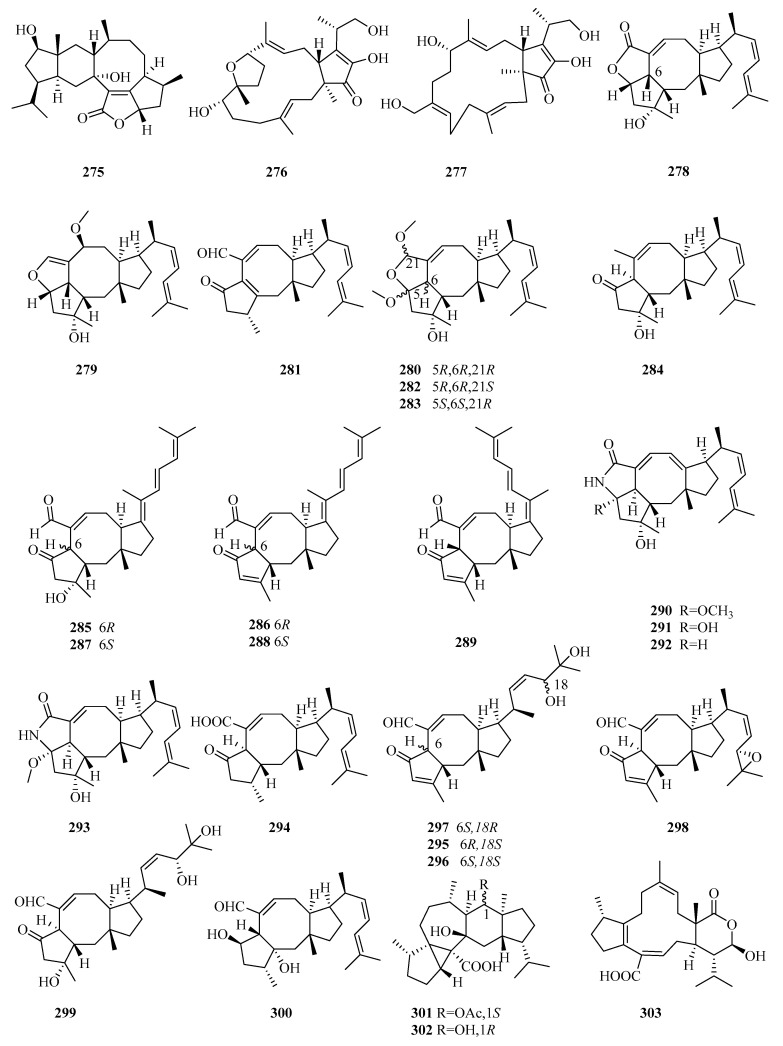
Chemical structures of sesterterpenes (**275**–**303**).

Sesterterpenoids are a small group of terpenoids with bioactivities in marine fungi. The number of newly isolated sesterterpenoids from marine fungi notably increased in the last five years (29 vs. 10 in 2011–2014, with 16 before 2010) (**275**–**303** in Figure 20) [7,8]. *Aspergillus* (25 compounds) is the main source of sesterterpenes from marine fungi, especially ophiobolins (23 compounds), and has always displayed cytotoxicity, anti-inflammatory activity, and enzyme inhibitory activity (e.g., against protein tyrosine phosphatase B). 

An investigation of the EtOAc extract of the fermentation cultures of the algicolous fungus *Alternaria alternata* k21-1, collected from the surface of the marine red alga *Lomentaria hakodatensis*, led to the discovery of sesteralterin (**275**), which was the first nitidasane sesterterpene naturally produced by fungi [80]. Sesteralterin displayed weak to moderate activity against three marine phytoplankton strains [80]. Two new oxidative terpestacin-type sesterterpenes, terpestacin B and 21-hydroxyterpestacin (**276** and **277**), and their biosynthetic precursor terpestacin, were isolated from the marine fungal strain *Arthrinium* sp. (5XNZ5-4) collected from the gut of a marine crab [81]. 

Marine sesterterpenes, especially ophiobolins, are mainly produced by *Aspergillus*. The ophiobolin family features a unique 5-8-5-tricarbocyclic skeleton whose biosynthesis involves five gene clusters responsible for C15, C20, C25, and C30 terpenoid biosynthesis [82]. Seven new ophiobolins, ophiobolins X–Z (**278**–**280**), 21-dehydroophiobolin U (**281**), 21*-epi-*ophiobolin Z and O (**282** and **283**), and 21-deoxyophiobolin K (**284**), along with 11 known analogs, were identified from the crude extracts of the liquid and solid cultures of the mangrove fungus *Aspergillus ustus* 094102 [83]. As evaluated by bioassays, compounds **280**, **282**–**284** and the known ophiobolins K, G, O, and Q, 6*-epi-*ophiobolin K, and 21,21-*O*-dihydro-6*-epi-*ophiobolin G displayed potential cytotoxic activity against six HTCLs (G3K, MCF-7, MD-MBA-231, MCF/Adr, A549, and HL-60), with IC_50_ values ranging from 0.6 to 9.5 μM [83]. The structure–activity relationships (SAR) study suggested that the 2,5-dimethoxyl-2H,3H,5H-furan moiety or H-6*β* and HO-3 are the pharmacophores responsible for the cytotoxicity against MCF-7 and MCF-7/Adr, while HO-3 and the *α*,*β*-unsaturated aldehyde moiety are important for MD-MBA-231 cytotoxicity, and the 2,5-dimethoxyl -2H,3H,5H-furan moiety is required for cytotoxicity against A549 and HL-60 [83]. 

Chemical investigations of the secondary metabolites of the marine fungus *Aspergillus flocculosus*, collected from the seaweed *Padina* sp. in Vietnam, resulted in the identification of five new ophiobolin-type sesterterpenes with a fully unsaturated side chain, 14,15-dehydro-6*-epi-*ophiobolins K and G (**285** and **286**), 14,15-dehydro-ophiobolins K and G (**287** and **288**), and 14,15-dehydro-(*Z*)-14-ophiobolin G (**289**), together with four known ophiobolins [84]. All isolated metabolites (compounds **285**–**289**, 6*-epi-*ophiobolins C and N, and ophiobolins C and N) showed potent cytotoxicity against six HTCLs (HCT-15, NUGC-3, NCI-H23, ACHN, PC-3, and MDA-MB-231) (GI_50_ = 0.14–2.01 μM) [84]. 

The bioassay and HPLC–UV-guided investigation of the fermentation culture of the mangrove endophytic fungus *Aspergillus* sp. ZJ-68 led to the identification of 11 new ophiobolin-type sesterterpenoids, asperophiobolins A–K (**290**–**300**), together with 12 known analogs, among which asperophiobolins A–D (**290**–**293**) were the first examples of a *γ*-lactam ring between C-5 and C-21 in ophiobolins [85]. According to the bioactivity assays, compounds **297**–**299**, ophiobolins O, U, and U’, and 21-deoxo-21-hydroxy-6*-epi-*ophiobolin G showed potential inhibitory activity of LPS-induced NO production in RAW 264.7 macrophage cells (IC_50_ = 9.6–25 μM vs. 38 μM for indomethacin), and compounds **291**, **293**, **294**, and **298**, ophiobolins G, P, and 21-deoxo-21-hydroxy-6*-epi-*ophiobolin G exhibited moderate inhibitory effects on the *Mycobacterium tuberculosis* (Mtb) protein tyrosine phosphatase B (MptpB) (IC_50_ = 24–42 μM, vs. 22 μM for oleanolic acid), particularly **297,** which showed significant anti-MptpB activity (IC_50_ =19 μM) [85]. 

Many new activities were reported for known ophiobolins. Most of ophiobolins show the significant inhibitory activity of tumor cell proliferation and are considered potential antitumor drugs [86]. MHO7 (6*-epi-*ophiobolin G) can suppress breast cancer cells by downregulating estrogen receptor alpha (ER*α*), which acts as a novel estrogen receptor degrader [87]. Further research of MHO7 showed that it is appropriate for oral administration according to the target reproductive organs and influences the metabolic pathways via short-chain fatty acids by regulating the levels of the gut microbiome (Ruminococcaceae and Lachnospiraceae) [88]. Another study was also carried out on the antitumor drug candidate ophiobolin O. The results showed that the compound could exert significant antitumor activity, with little in vivo and in vitro toxicity, by inducing G1 phase arrest in the human breast cancer MCF-7 cells related to the AKT/GSK3*β*/cyclin D1 signaling pathway [89].

Two new unusual 5/3/7/6/5-pentacyclic sesterterpenes (aspterpenacids A and B) (**301** and **302**) [90] and a new atypical 5/12/6-tricyclic sesterterpene acid (bipolarenic acid, **303**) [91] were isolated from the two mangrove fungi (*Aspergillus terreus* H010 and *Lophiostoma bipolare* BCC25910) but were devoid of antibacterial activity or cytotoxicity. 

### 3.5. Triterpenes

**Figure 21:**
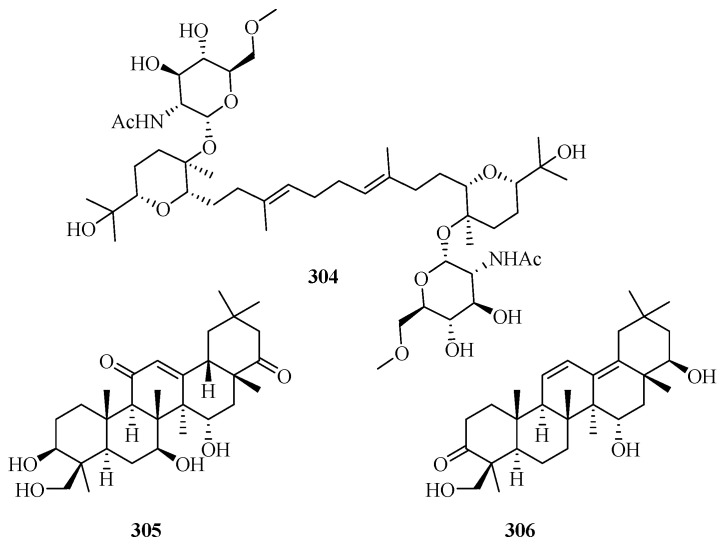
Chemical structures of triterpenes (**304**–**306**).

Triterpenes (excluded from steroids) are very rare in marine fungi, and only 11 new compounds were discovered in recent years (including three new molecules, **304**–**306**, in 2015–2019, as shown in Figure 21) [7,8].

The novel triterpene glycoside, auxarthonoside (**304**), possessing a rare sugar moiety (*N*-acetyl-6-methoxy-glucosamine) in nature, was reported in the marine sponge-derived fungus *Auxarthron reticulatum* [92]. The Red Sea hard coral-derived fungus *Scopulariopsis* sp. yielded two new triterpenoids (3*β*,7*β*,15*α*,24-tetrahydroxyolean-12-ene-11,22-dione and 15*α*,22*β*,24-trihydroxyolean-11,13-diene-3-one) (**305** and **306**) and three known derivatives, 7*β*,15*α*,24-trihydroxyolean-12-ene-3,11,22-trione, 15*α*,24-dihydroxyolean-12-ene-3,11,22-trione, and soyasapogenol B—none of which displayed significant cytotoxic, antibacterial, or antitubercular activities [93].

### 3.6. Meroterpenes

Fungal meroterpenoids can be classed into two major groups according to their biosynthetic origins as polyketide–terpenoids (>80% fungal meroterpenoids, including orsellinic acid, 5-methylorsellinic acid (5-MOA), and 3,5-dimethylorsellinic acid (DMOA), and 6-methylsalycylic acid (6-MSA) as the polyketide part) and non-polyketide–terpenoids (including indole diterpenoids and shikimate-derived terpenoids, such as tricycloalternarenes). Interesting, these two groups of meroterpenes are found in marine fungi. Simple isopentenyl (C5)-substituted alkaloids, polyketides, peptides, and shikimates, and their derivates, were excluded from this review. 

**Figure 22:**
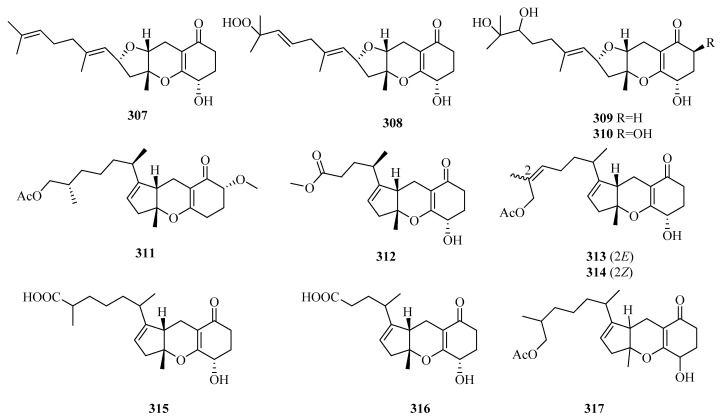
Chemical structures of sesquiterpenes (**307**–**317** from *Alternaria* sp.).

**Figure 23:**
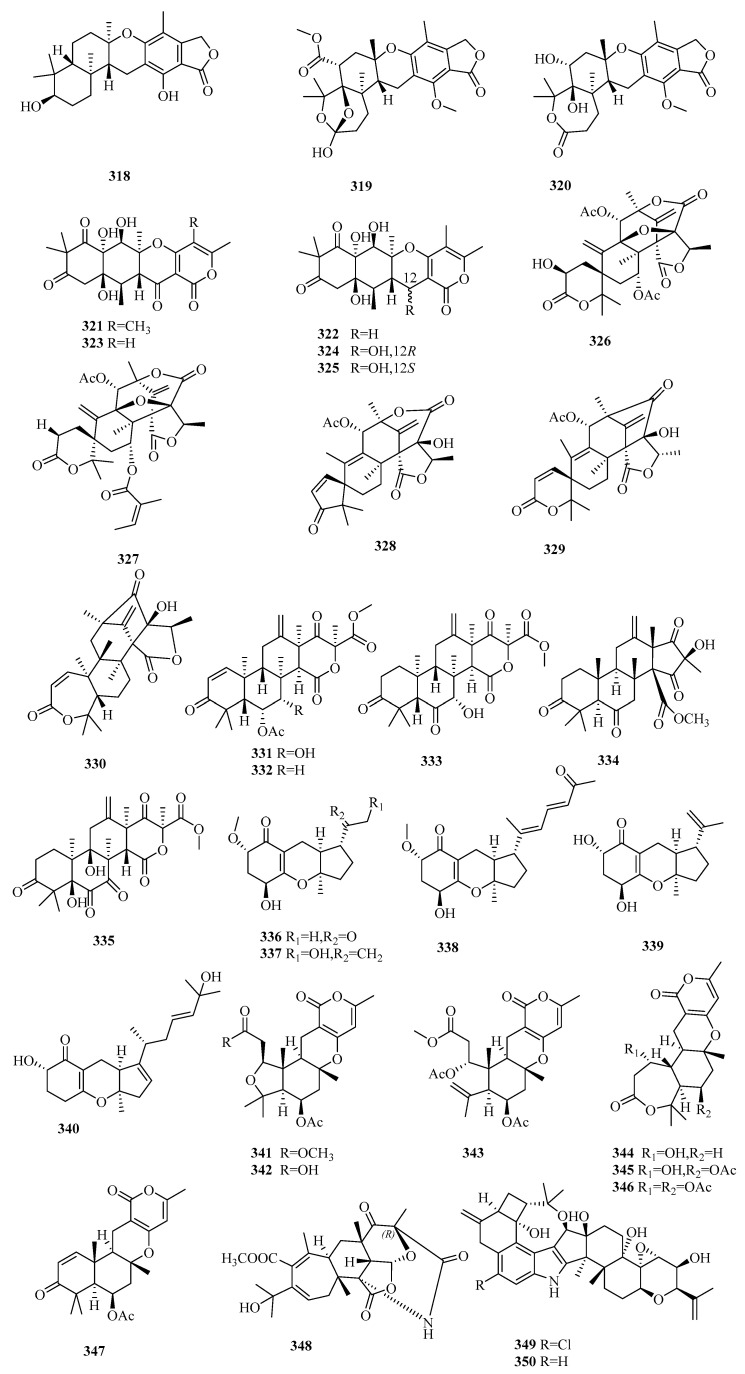
Chemical structures of meroterpenes (**318**–**350** from *Aspergillus* sp.).

**Figure 24:**
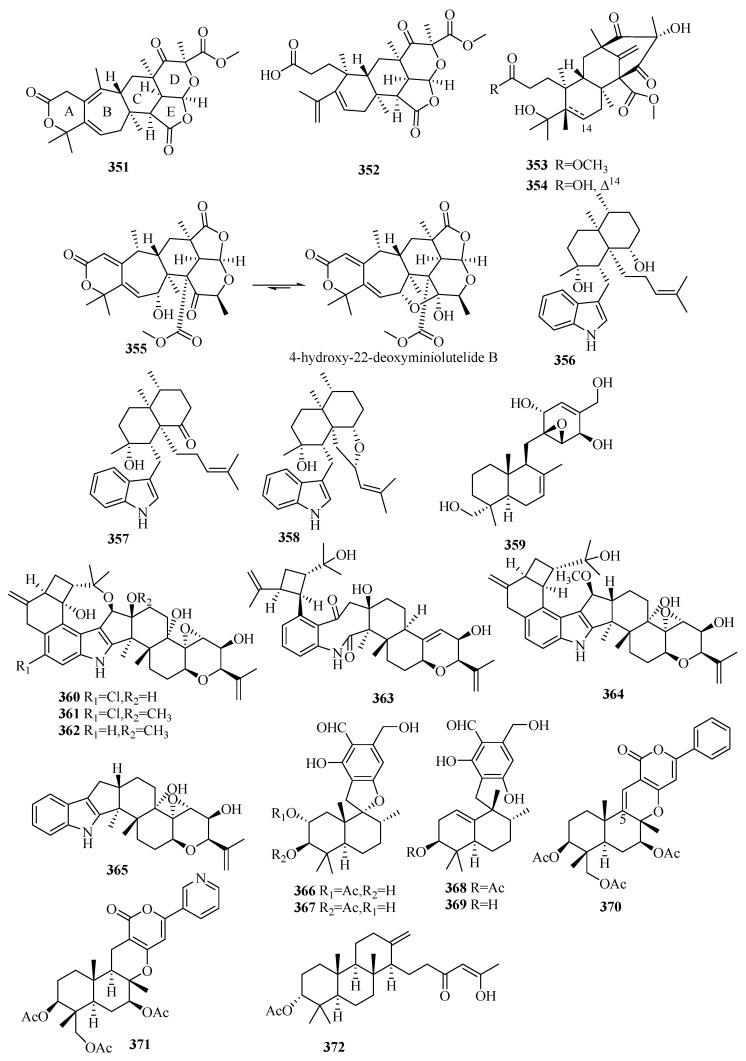
Chemical structures of meroterpenes (**351**–**358** from *Eupenicillium* sp., **359** from *Lophiostoma* sp., **360**–**365** from *Mucor* sp., **366**–**369** from *Myrothecium* sp., and **370**–**372** from *Neosartorya* sp.).

**Figure 25:**
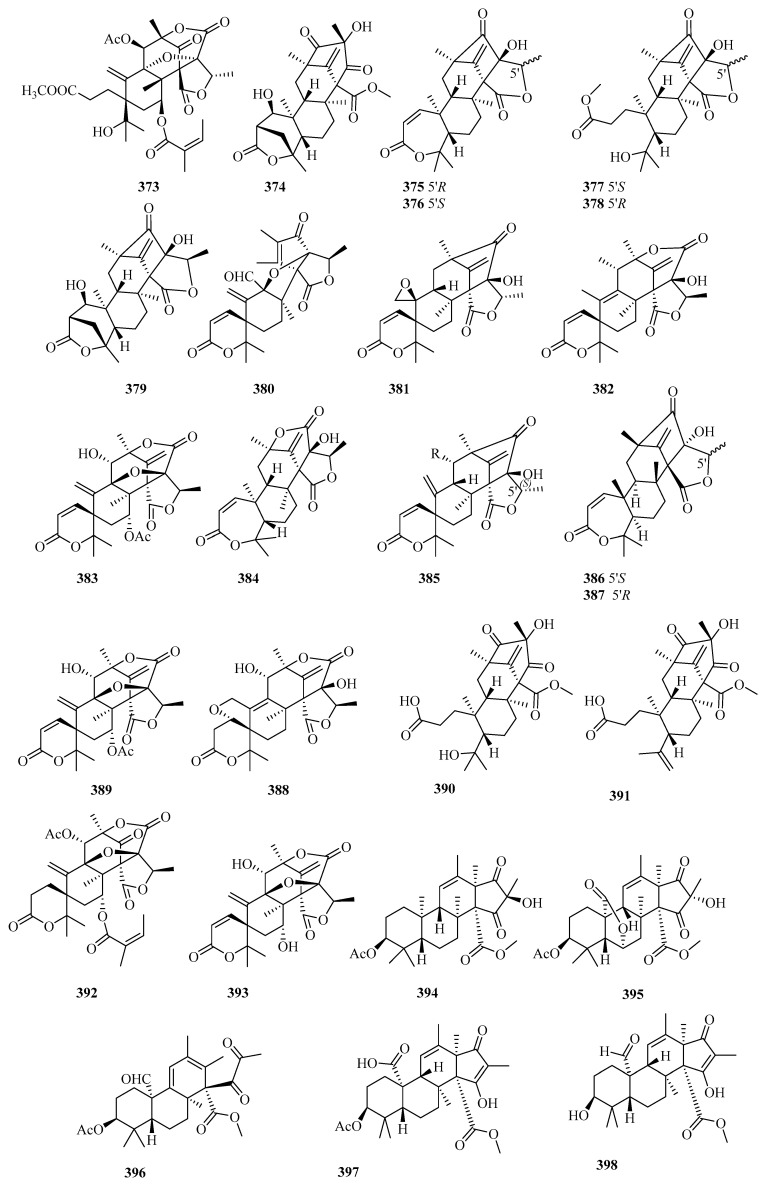

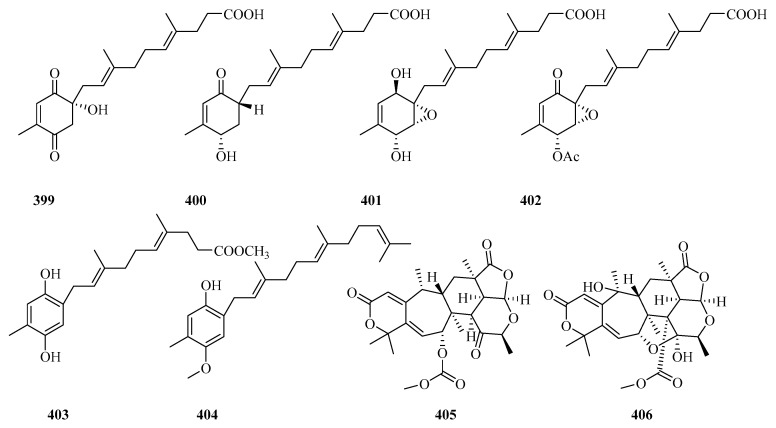
Chemical structures of meroterpenes (**373**–**406** from *Penicillium* sp.).

**Figure 26:**
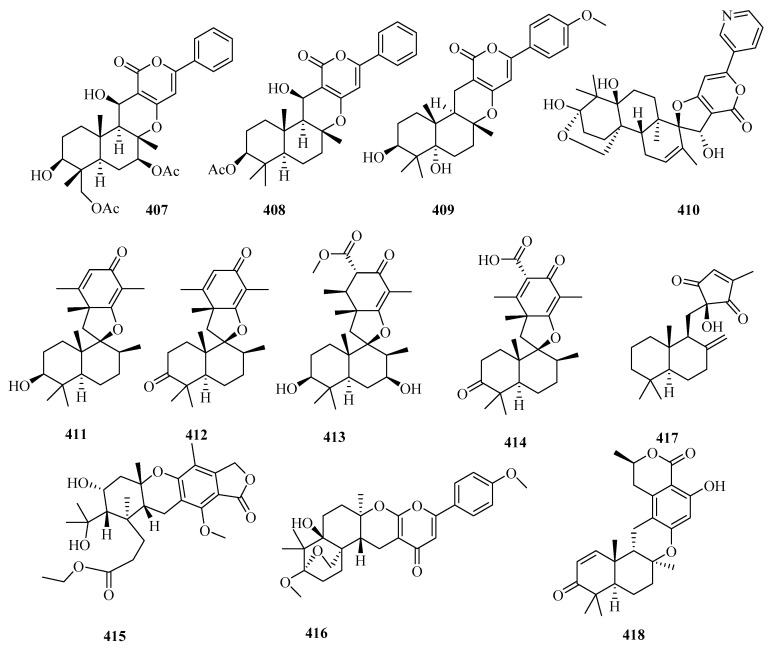

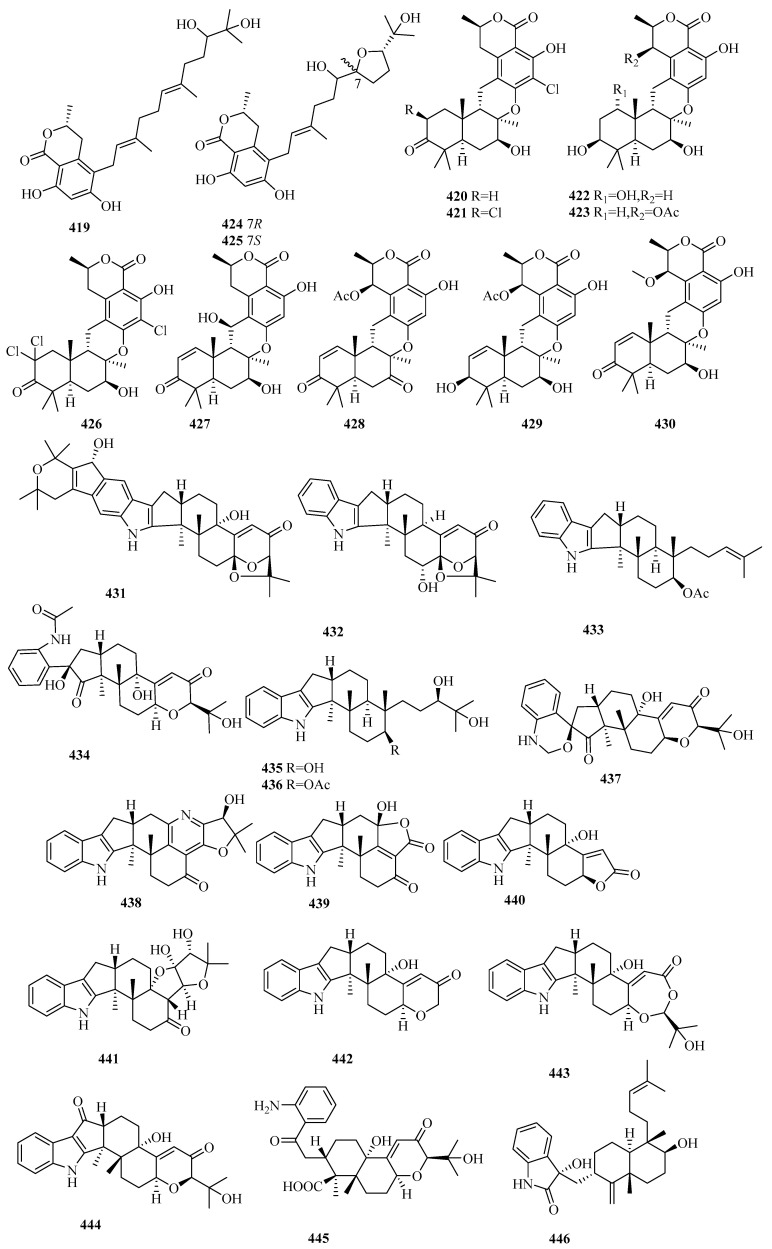
Chemical structures of meroterpenes (**407**–**446** from *Penicillium* sp.).

**Figure 27:**
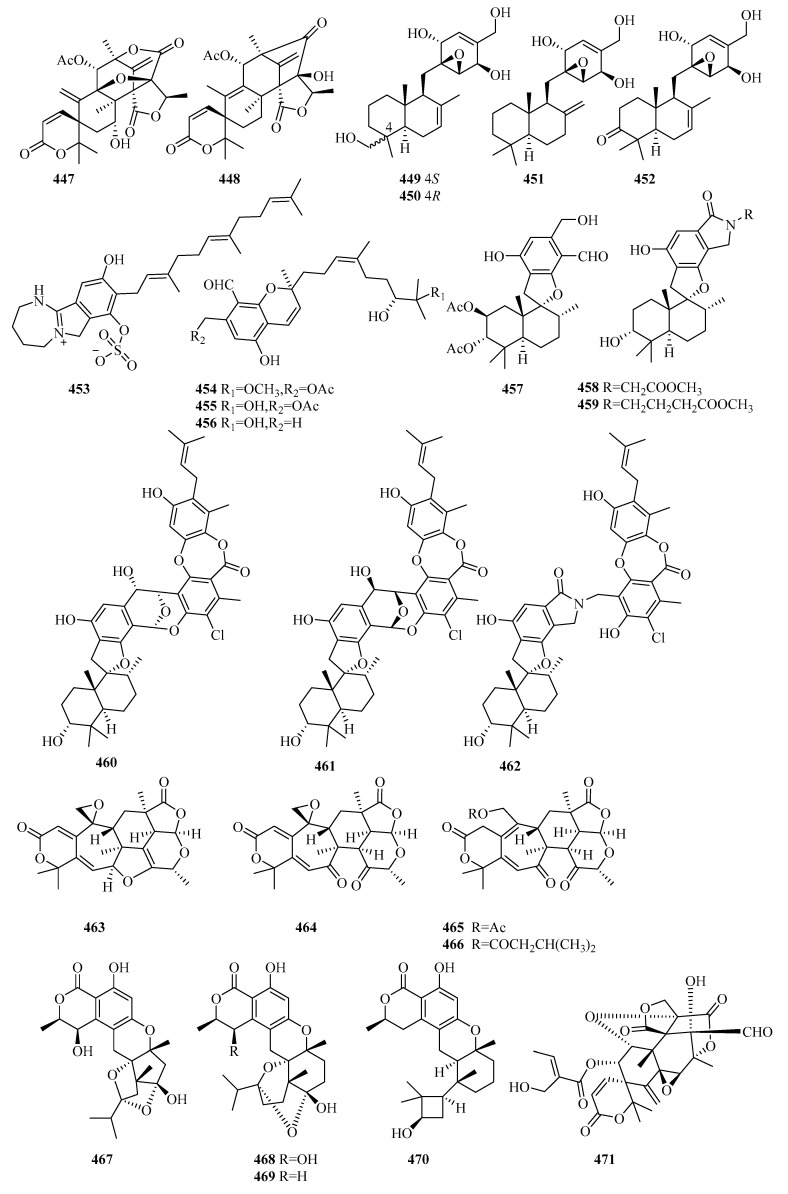
Chemical structures of meroterpenes (**447**–**448** from *Pestalotiopsis* sp., **449**–**452** from *Pleosporales* sp., **453**–**462** from *Stachybotrys* sp., and **463**–**471** from *Talaromyces* sp.).

There are 165 novel meroterpenoids (**307**–**471**, Figure 22, Figure 23, Figure 24, Figure 25, Figure 26 and Figure 27). Polyketide-terpenoids (the polyketide part is mainly derived from DMOA) and indole diterpenoids (27, 16.4%) were largely discovered in marine fungi during the last five years. *Penicillium* (75, 45.5%) and *Aspergillus* (33, 20.0%) were the principal sources of marine meroterpenoids, though they were also found in 10 other genera of marine fungi (*Alternaria*, *Eupenicillium*, *Lophiostoma*, *Mucor*, *Myrothecium*, *Neosartorya*, *Pestalotiopsis*, *Pleosporales*, *Stachybotrys*, and *Talaromyces*). These fungi were mainly isolated from living matter (125, 75.8%), such as marine animals (66, 40.0%) and aquatic plants (59, 35.8%), and to a lesser extent, living environments (35, 21.2%, mainly marine sediments). The most important specific source of fungi was algae (31), followed by sponges (24), mangrove habitats (23), other marine sediments (18), deep-sea sediments (11), mollusks (12), and sea worms (11). According to the bioactivity assessment, 37% of the new meroterpenoids (98 actives in 265 test compounds) displayed various activities, such as enzyme inhibitor activity (17%, 17), anti-inflammatory activity (16%,16), lethal toxicity activity (16%,16), antibacterial activity (15%, 15), cytotoxicity (13%, 13), and antiviral activity (10%, 10). 

#### 3.6.1. *Alternaria* sp.

Eight new tricycloalternarene (TCA)-type meroterpenes, tricycloalterfurenes A–D (**307**–**310**) [80], 17-*O*-methyltricycloalternarene D, and methyl nortricycloalternarate (**311** and **312**) [94]; (2*E*)-TCA 12a and (2*Z*)-TCA 12a (**313** and **314**) [95]; and four known congeners, TCAs F, D, 1b, and 11a, were isolated from the cultures of two marine red alga-epiphytic fungi *Alternaria alternate* (k21-1 and k23-3). These compounds showed weak or moderate inhibition of marine plankton species. Among them, compounds **307**–**310** possessed a rare tetrahydrofuran ring in TCAs [80]. Two new TCA acids (tricycloalternarenes K and L) (315 and 316) [96] and a new TCA ester (2*H*-(2*E*)-tricycloalternarene 12a, **317**) [97] and six known derivatives were discovered from two fungal strains, *Alternaria alternata* ICD5-11, associated with marine isopod *Ligia exotica*, and *Alternaria* sp. W-1, derived from *Laminaria japonica*, respectively. In the bioassay experiments, compound **317** and TCA-3a exhibited weak cytotoxicity against SMMC-7721 cells in a dose-dependent manner (IC_50_ = 49.7, 45.8 µg/mL) [97]. Further studies revealed that the anticancer mechanism of TCA-3a is related to cell cycle arrest in the G1 phase, and that induction of apoptosis involved two pathways (mitochondrial and death receptor pathways) [97]. 

#### 3.6.2. *Aspergillus* sp.

Three new austalide-type meroterpenoids, austalides S–U (**318**–**320**), were purified and identified from the sponge-derived fungus *Aspergillus aureolatus* HDN14-107, among which **318** possesses a 5/6/6/6/6-pentacyclic ring system with the *trans* configuration of C-11 [98]. Only compound **318** exhibited marked inhibitory effects of anti-influenza virus A (H1N1) activity with IC_50_ values of 90 µM (ribavirin, IC_50_ 102 µM) but without cytotoxicity (IC_50_ > 50 µM) [98].

The marine algal-associated fungus *Aspergillus* sp. ZL0-1b14 yielded four new highly oxygenated putative triketide-sesquiterpenoid drimane-type meroterpenes, aspertetranones A–D **(321**–**324**), featuring an unusual skeleton rearrangement in the terpenoid part [99]. Using LPS-stimulated RAW264.7 as the anti-inflammatory cell model, compounds **321** and **324** inhibited the production of IL-6 and IL-1*β* in a dose-dependent manner (43%/42% and 69%/47% inhibition rates of IL-6/IL-1*β* at 40 μM, respectively), while compounds **322** and **323** only showed weak inhibitory effects [99]. A new enantiomer of compound **324**, 12*-epi-*aspertetranone D (**325**), was isolated from marine sediment-derived *Aspergillus flocculosus* and showed 41% inhibition against the colony formation of 22Rv1 prostate cancer cells at 100 μM but without cytotoxicity [100].

The chemical investigation of the mangrove endophytic fungus *Aspergillus* sp. 16-5c led to the isolation of ten DMOA-derived austin-type meroterpenoids, including the novel 2-hydroacetoxydehydroaustin (**326**) [101]. The stereochemistry configurations of known compounds, preaustinoid A2, isoaustinol, dehydroaustin, and dehydroaustinol, were first determined by XRD, and the latter three molecules displayed anti-acetylcholinesterase (anti-AchE) activity (IC_50_ = 0.4~3 µM) [101]. An unusual austinoid, 1,2-dehydro-terredehydroaustin (**327**), from the mangrove-derived fungus *Aspergillus terreus* H010, showed weak anti-inflammatory (NO) effects in RAW 246.7 cells (IC_50_ 42.3 μM vs. 30.7 μM for indomethacin) [102]. The marine endophytic fungus of the brown alga, *Aspergillus* sp. ZYH026, was able to yield structurally diverse DMOA-derived meroterpenoids, including three new asperaustins A–C (**328**–**330**), and the stereostructures of the known austinoneol A and precalidodehydroaustin were ascertained [103]. Compound **328** possesses a special spiro[4.5]deca-3,6-dien-2-one unit with an unusual 5/6/6/6/5 pentacyclic skeleton; **329** and **330** feature a spiro-lactone core and a heptatomic lactone ring, respectively [103]. None of these compounds showed AChE inhibitory activity [103].

Terretonins, a group of polyoxygenated meroterpenoids mostly possessing the same unique tetracyclic scaffolds and primarily isolated from the *Aspergillus* genus, are derived from DMOA and farnesyl pyrophosphate (FPP) [104]. Three new terretonins with a reversed orientation at H-14, terretonins H and I (**331** and **332**) [105], along with terretonin D1 (**333**) [106] were obtained from the marine sediment-derived fungus *Aspergillus ustus* KMM 4664 and the marine fungus *Aspergillus terreus* EN-539 associated with the fresh gut of pacific oyster, respectively. Compounds **331** and **332** displayed weak inhibition of fertilized sea urchin eggs [105], and **333** was weakly anti-inflammatory (NO) in RAW264.7 cells [106]. In an alternative study, two new terretonin derivatives, aperterpenes N (**334**) and O (**335**), and two known analogs were reported in the marine algal-derived fungus *Aspergillus terreus* EN-539, among which the stereostructures of the known terretonins A and G were first established by XRD [107]. Compound **334** exhibited inhibitory effects in influenza neuraminidase (IC_50_ = 18.0 nM vs. 3.2 nM for oseltamivir), and terretonin G displayed weak inhibition of *Micrococcus luteus* and *Staphylococcus aureus* growth (MIC = 8–32 μg/mL vs. 1.0 μg/mL for chloramphenicol) [107].

Five new TCA-type meroterpenoids, guignardones J–M (**336**–**339**) [108] and tricycloalternarene 14b (**340**) [109], along with eight known analogs, were isolated and identified from the mangrove endophytic fungus *Aspergillus flavipes* AIL8 and one symbiotic strain, *Aspergillus* sp. D, from the coastal plant *Edgeworthia chrysantha* Lindl. Only **340** possessed weak antimicrobial effects on three human pathogenic strains (*Escherichia coli*, *Staphylococcus aureus*, and *Candida albicans*) (MIC ≥ 15.63 µM) and moderate cytotoxic effects against A-549 cells (IC_50_ = 8.89 µM) [109].

Two previously undescribed pyrone meroterpenoids featuring an uncommon 5/6/6/6- tetracyclic skeleton with a tetrahydrofuran ring, asperversins A (**341**) and B (**342**), together with five new derivatives (asperversins C–G, **343**–**347**) and a known asperdemin, were obtained from the marine sediment-derived fungus *Aspergillus versicolor* [110]. After assessing the inhibitory activity of acetylcholinesterase enzyme (AChE) and the cytotoxicity of all metabolites, only **347** displayed a moderate anti-AChE effect (IC_50_ = 13.6 µM vs. 3.57 µM for galanthamine) [110].

An unusual stress metabolite induced by Co, aspergstressin (**348**), possessing a unique fused polycyclic structure, was isolated from the culture broth of the fungus *Aspergillus* sp. WU 243, collected from a crab dwelling in heavy metal-rich hydrothermal vents [111].

Two new indole diterpenoids, 19-hydroxypenitrem A (**349**) with Cl-substitution and 19-hydroxypenitrem E (**350**), were sourced from the cultures of the endophytic fungus *Aspergillus nidulans* EN-330 collected from marine red alga *Polysiphonia scopulorum* var. villum [112]. Compounds **349**–**350** exhibited potent brine shrimp cytotoxic activity (LD_50_ = 3.2–4.6 μM vs. 10.7 μM for colchicine) [112], and compound **349** showed moderate antimicrobial activity against four human-pathogens (*Edwardsiella tarda* and *Vibrio anguillarum*) and aqua-pathogens (*Escherichia coli* and *Staphylococcus aureus*) (MIC = 16–32 μg/mL), while **350** had weak antibacterial activity against two bacteria (*E. tarda* and *E. coli*) [112]. The above SAR experiments suggest that Cl-substitution at C-6 enhanced the cytotoxicity of brine shrimp and antimicrobial activity, while 19-OH substitution diminished this activity [112].

#### 3.6.3. *Eupenicillium* sp.

The LC-MS and ^1^H NMR-based investigation of the fermentation culture of the marine sponge-associated fungus *Eupenicillium* sp. 6A-9 resulted in the discovery of ten meroterpenoids, including two rare 6(D)/5(E) ring-fused DMOA-related meroterpenoids, eupeniacetal A (**351**) and eupeniacetal B (**352**), and three new meroterpenoids, 1-methoxy-hydropreaustinoid A1 (**353**), hydroberkeleyone B (**354**), and 22-deoxy-10-oxominiolutelide B (**355**) [113]. Compound **355** and the known 22-deoxy-miniolutelide B exist as an equilibrium mixture [113]. All tested compounds (**351**–**355**) showed dramatic immune-suppressive activity towards TNF-*α* production in the LPS-induced THP-1 cell line (IC_50_ = 22.6–43.1µM vs. 0.23 µM for pomalidomide) but were inactive against HTCLs and pathogenic bacteria [113].

The cytotoxicity-guided investigation of the fermented crude extract of *Eupenicillium* sp. HJ002, an endophytic fungus of mangrove, yielded three new indole diterpenes, penicilindoles A–C (**356**–**358**) [114]. Compounds **356** and **357** were cytotoxic towards three HTCLs (A549, HeLa, and HepG2) (IC_50_ = 1.5–47.2 μM), among which **356** showed potential cytotoxicity against A549 and HepG2 (IC_50_ = 5.5 and 1.5 μM, respectively) [114].

#### 3.6.4. *Lophiostoma* sp.

One new merosesquiterpenoid, craterellin D (**359**), and a known analog craterellin A were isolated from a soft coral-derived fungus, *Lophiostoma* sp. ZJ-2008011, from which the absolute configuration of craterellin A was first determined [115]. In the bioassays of **359**, craterellin A, and the acetonide and acetylation products of craterellin A, only craterellin A showed moderate antibacterial activity against *Bacillus cereus*, *Escherichia coli*, *Staphylococcus aureus*, and *Micrococcus luteus* (MIC = 3.12–6.25 μM), indicating that the OH in the hydroquinone scaffold may decrease antibacterial activity [115].

#### 3.6.5. *Mucor* sp.

The genome mining-aided chemical investigation of the mangrove fungus *Mucor irregularis* QEN-189 resulted in the isolation of 20 structurally diverse indole diterpenes, including six novel compounds, rhizovarins A–F (**360**–**365**) [116]. Compounds **360**–**362** were elucidated as an unusual 4,6,6,8,5,6,6,6,6-fused indole diterpene moiety scaffold, including the rarely observed acetal connected to hemiketal (**360**) or ketal (**361** and **362**) functionality [116]. Compounds **360** and **361**, penitrems A, C, and F; and 3b-hydroxy-4b-desoxypaxilline (a paxilline-type indole diterpene) showed activity against two HTCLs (A-549 and HL-60) (IC_50_ = 2.6–11.5 μM), while compound **364** exhibited activity only against A-549 (IC_50_ = 9.2 μM), implying that chlorine substitution might be required for the cytotoxicity of these target cells [116].

#### 3.6.6. *Myrothecium* sp.

Studies of the fungus *Myrothecium* sp. OUCMDZ-2784 associated with the salt-resistant medicinal plant, *Apocynum venetum* (Apocynaceae) (the estuary of Yellow River, China), afforded four new meroterpenoids, myrothecisins A–D (**366**–**369**), which displayed a weak *α*-glucosidase inhibitory effect (IC_50_ = 0.50, 0.66, 0.058, and 0.20 mM vs. 0.47 mM for acarbose) [117].

#### 3.6.7. *Neosartorya* sp.

Bioactivity-guided isolation of the fermentation metabolites of the marine fungus *Neosartorya pseudofischeri*, which was separated from the inner tissue of the starfish *Acanthaster planci*, yielded two new meroterpenes, 5-olefin phenylpyropene A (**370**) and 13-dehydroxylpyripyropene A (**371**), together with four known analogs, phenylpyropene A and C, pyripyropene A, and 7-deacetylpyripyropene A [118]. The six isolated compounds showed obvious cytotoxicity towards the insect cell line Sf9 (the fatality rate was 85.2–95.0% after 48 h of compound treatment at 50 mg/L) [118]. From the crude secondary metabolite extract of the algicolous fungus *Neosartorya takakii* KUFC 7898 collected from marine algal *Amphiroa* sp. (Samaesarn Island, Thailand), a new triketide-type meroditerpene, sartorenol (**372**), was obtained, though **372** lacked antibacterial activity and quorum-sensing inhibition [119].

#### 3.6.8. *Penicillium* sp.

In the antibiotic screening experiments, a new metabolite-austinone (**373**) and six known austin derivatives were discovered from the fungi *Penicillium* sp. Y-5-2 collected from the sediment of a hydrothermal vent (Kueishantao, Taiwan, China) [120].

Based on the fungal genome cluster analysis, a sponge-associated fungus strain, *Penicillium brasilianum* WZXY-m122-9, was selected to search for diverse and novel meroterpenoids because of the highly similar *aus’* clusters. Twelve new DMOA-related meroterpenoids, brasilianoids A–F (**374–379**) [121] and G–L (**380**–**385**) [122], and 12 known analogs, were disclosed. Notably, brasilianoids A (**374**) and F (**378**), possessing an unprecedented motif with a *γ*-lactone in ring A; brasilianoids B–C (3**76**–**376**), bearing a 7/6/6/5/5-pentacyclic skeleton [121]; brasilianoid G (**380**), characterized by a rare 6/6/5/5/5 pentacyclic scaffold; and brasilianoid K (**384**), featuring an uncommon 7/6/6/6/5 pentacyclic system [122] were first reported in nature. Compound **374** significantly promoted the expression of filaggrin and caspase-14 in HaCaT cells in a dose-dependent manner without cytotoxicity, suggesting that it could be used to reduce UVB-induced cell damage, while compounds **375** and **376** showed moderate anti-inflammatory activity (NO) in RAW 264.7 cells (IC_50_ = 37.69/33.76 μM) [121]. Compounds **376**–**378** displayed very weak antiviral (HBV) activity [121]. After testing for multiple activities (including cytotoxicity and antibacterial activity), the compounds brasilianoid L (**385**), austinol, and dehydroaustin (three different scaffolds) were all found to significantly inhibit bacteria-infected host cells by preventing the polymerization of actin in RAW264.7 [122].

A pair of new epimeric austin-type pentacyclic lactones, preaustinoids E (**386**) and F (**387**), were reported in the marine sediment-derived fungus, *Penicillium* sp. FCH061 (Chuja-do, Korea), but were devoid of cytotoxicity and antimicrobial or enzyme inhibitory activity [123]. The ^1^H NMR facilitated the isolation of marine fungal *Penicillium* sp. SF-5497 (sea sand, Gijang-gun, Busan, Korea), which led to the obtaining four new DMOA-derived meroterpenoids, furanoaustinol and 7-acetoxydehydroaustinol (**388** and **389**) [124], and the preaustinoids A6 and A7 (**390** and **391**) [125] together with 11 known derivatives. Furanoaustinol (**388**) possesses an extra tetrahydrofuran ring that forms an uncommon hexacyclic austin skeleton [124]. Compounds **388** and **390** and berkeleyone C showed weak or moderate dose-dependent anti-PTP1B activity (IC_50_ = 77.2, 17.6, 58.4 μM, respectively) [124,125], and **389** weakly inhibited the overproduction of NO in LPS-induced BV2 microglial cells with an IC_50_ value of 61.0 μM [124]. Two new DMOA-type meroterpenoids, penicianstinoids A and B (**392** and **393**), together with seven known analogs were uncovered from the mangrove endophytic fungus *Penicillium* sp. TGM112, among which **392** possesses a rare carbonyl group at C-1′-C-2′ in the austinoids [126]. Moreover, the stereostructures of furanoaustinol (**388**) and 1,2-dihydro-7-hydroxydehydroaustin were reported [126]. Compounds **392** and **393**, austinol, and austin displayed toxicity against newly hatched larvae of *Helicoverpa armigera* Hubner (IC_50_ = 50–200 μg/mL vs. 25 μg/mL for azadirachtin), and compounds **392**, **393**, 7-hydroxydehydroaustin, and dehydroaustinol showed inhibition against *Caenorhabditis elegans* (EC_50_ = 9.4–38.2 μg/mL vs. 4.8 μg/mL for levamisole) [126].

From the deep-sea-derived fungus *Penicillium allii-sativi* (deep seawater, western Pacific), we isolated two new tetraketide-type merosesquiterpenes, andrastone A (**394**) and 16*-epi-*citreohybriddione A (**395**), and the already known citreohybriddione A (confirmed by XRD), among which **394** was a rare andrastin possessing an unusual cyclopentan-1,3-dione without the lactone moiety at C-23 [127]. Compound **394** displayed significant selective antiproliferative effects against HepG2 (IC_50_ = 7.8 µM) by direct caspase-8-mediated caspase-3 activation and regulating the RXR*α* pathways to induce apoptosis [127]. An analysis of the chemical pharmaceutical ingredient of fungal *Penicillium simplicissimum* MA-332, collected from the rhizospheric soil of the marine mangrove plant *Bruguiera sexangula* var. *rhynchopetala,* resulted in a new DMOA-type meroterpenoid, simpterpenoid (**396**), which is characterized by a highly functionalized cyclohexadiene motif bearing *gem*-propane-1,2-dione and methylformate groups [128]. Compound **396** showed significant influenza neuraminidase inhibitory activity at a nanomolar scale (IC_50_ = 8.1 nM vs. 3.2 nM for oseltamivir) and weak or absent antimicrobial activities (MIC ≥ 64 μg/mL) [128].

Citreohybridonol was re-isolated from the sponge-derived fungus *Penicillium atrovenetum* and found by NMR to exist as a keto–enol equilibrium tautomer in the solvent [129]. Then, the structure was assigned with the Flack parameter 0.06(3) in X-ray crystallography [129]. Citreohybridonol, re-obtained from the sponge-derived fungus *Toxicocladosporium* sp. SF-5699, was able to inhibit the production of NO and PGE2 and the expression of iNOS, COX-2, and other pro-inflammatory cytokines, including IL-1*β* and TNF-*α*, in the LPS-stimulated BV2 cells [130]. Further experiments demonstrated that citreohybridonol displayed anti-neuroinflammatory activity involved in regulating the TLR4/ MyD88-mediated NF-*κ*B and p38/MAPK inflammatory pathways [130].

Two new DMOA-derived meroterpenoids (15-deacetylated citreohybridone E and 3-deacetylated andrastin A) (**397** and **398**) [11], featuring a rare 23-aldehyde or 23-carboxylic acid group, and four new farnesylcyclohexenones (peniginsengins B–E, **399**–**402**) [131], possessing a highly oxygenated 1-methylcyclohexene (ambuic acid derivatives) unit linked with a farnesyl-derived carboxylic acid (C12) side chain rarely found in nature, and seven known analogs, were isolated from the deep-sea fungus *Penicillium* sp. YPGA11. Bioassays showed that **400**–**402** possessed moderate activity toward methicillin-sensitive *Staphylococcus aureus* (MIC= 8–32 μg/mL), while **400** and **402** exhibited an effect against methicillin-resistant *S. aureus* with MIC values of 32 and 64 μg/mL [131]. In another study, six farnesyl meroterpenes, including two new meroterpenoids, chrysogenester (**403**) and 5-farnesyl-2-methyl-1-*O*-methylhydroquinone (**404**), were observed in the jellyfish-derived fungus *Penicillium chrysogenum* J08NF-4 [132]. Interestingly, **403** was able to suppress the in vitro inflammatory response by participating in the peroxidase proliferator-activated receptor (PPAR-*γ*)/NF-*κ*B signalling pathway [132].

The combined chemical analysis strategies of HPLC–HRMS-based hierarchical clustering analysis (HCA) and MS/MS molecular networking were used to isolate the metabolites of the marine fungus *Penicillium ubiquetum* MMS330 from the blue mussel *Mytilus edulis* (Loire estuary, France) through six different culture media following the OSMAC approach [133]. Two new meroterpenoids, 22-deoxyminiolutelide A (**405**) and 4-hydroxy-22-deoxyminiolutelide B (**406**), together with seven known analogs, were selectively overexpressed and obtained from a seawater CYA (Czapek yeast extract agar) medium and were devoid of cytotoxicity at 50 μM (KB and MCF-7 cells) [133]. Additionally, 22-deoxy-10-oxominiolutelide B was found to easily transform into either compound **405** or 22-deoxyminiolutelide B [133]. Thus, the latter two compounds may be artefacts [133].

Phenylpyropenes E (**407**) and F (**408**) and seven known phenylpyropenes and pyripyropenes, were characterized from the marine-derived fungus *Penicillium concentricum* ZLQ-69 (seawater, Bohai Sea coast, China) [134]. In the bioassay of the three strains of HTCLs, only **407** and phenylpyropene C showed moderate cytotoxicity toward MGC-803 cells (IC_50_ = 19.1 and 13.6 μM, respectively) [134].

The mangrove endophytic fungus *Penicillium* sp. SK5GW1L, collected from the leaves of *Kandelia candel* (Shankou, Guangxi, China), was found to produce five *α*-pyrone meroterpenoids, including one new 3-epiarigsugacin E (**409**), of which only **409** weakly inhibited AchE, while arisugacin B, territrem C, and terreulactone C, all already known, showed potent anti-AchE activity (IC_50_ = 0.03–3.03 μM) [135].

A new meroterpenoid, 15-hydroxydecaturin A (**410**), and seven other analogs were obtained from the marine algae-derived fungus *Penicillium oxalicum* EN-290 (*Codium fragile*, Qingdao, China) [136]. Four new spiromeroterpenoids, chermesins A–D (**411**–**414**), characterized by a drimane-type sesquiterpene moiety with a rare cyclohexa-2,5-dienone unit, were obtained from the fungus *Penicillium chermesinum* EN-480, which was separated from the marine red alga *Pterocladiella tenuis* (Rongcheng, China). Compounds **411** and **412** weakly or moderately inhibited the pathogens *Micrococcus luteus, Candida albicans, Escherichia coli*, and *Vibrio alginolyticus* (MIC = 8–64 μg/mL) [137].

A new austalide-type meroterpenoids, austalide H acid ethyl ester (**415**) [138], was isolated from the marine algae-derived fungi *Penicillium thomii* Maire KMM 4645, and compound **415** showed inhibition of the enzyme *endo-*1,3-*β*-d-glucanase [138]. Another deep-sea-derived fungus, *Penicillium chrysogenum* (SCSIO 41001), produced a new merosesquiterpenoid, yaminterritrem C (**416**), bearing a novel naphtho[2,1-b]pyrano-[3,2e]pyran scaffold [139]. In the screen for renoprotective agents from the deep-sea-derived fungus *Penicillium* sp. F00120, a new meroterpene was discovered, penicilliumin B (**417**), which possesses an unusual drimane sesquiterpene methylcyclopentenedione motif possibly from sesquiterpene quinones [140]. Compound **417** showed promising potential renoprotective activity with low toxicity by inhibiting the kidney fibrogenic action of high glucose in rat glomerular mesangial cells (RMC) involved in the disruption of oxidative stress [140].

Chrodrimanins, a small subclass of meroterpenoids sharing a 6/6/6/6/6 pentacyclic ring scaffold, were derived from a drimane-type sesquiterpene and a C10 polyketide (shown as a 6,8-dihydroxy-3-methylisochroman-1-one or a 3,6,8-trihydroxy-3,4dihydronaphthalen-1-one moiety). Five chrodrimanin-type meroterpenes, including the new verruculide A (**418**) and the biosynthetic precursor verruculide B (**419**) with a linear sesquiterpene moiety, together with three known analogs, were isolated from Indonesian ascidian-derived *Penicillium verruculosum* TPU1311. Compound **418** and chrodrimanins A and H showed good anti-PTP1B activity (IC_50_ = 8.4–14.9 μM), while **419** weakly inhibited PTP1B [141]. In an alternate study, 11 new chrodrimanin-type meroterpenes, chrodrimanins K–N (**420**–**423**), the biosynthetic precursors verruculides B2 (**424**) and B3 (**425**) [142], chrodrimanins O–S (**426**–**430**) [143], and eight known analogs were obtained from the fungus *Penicillium* sp. SCS-KFD09 isolated from the marine worm *Sipunculus nudus* (Haikou Bay, China). Among them, chrodrimanins K (**420**) and L (**421**) correspond to two unusual chlorinated chrodrimanins. Chrodrimanin O (**426)** features special dichlorine functionality as an uncommon trichlorinated meroterpenoid, and the absolute configurations of chrodrimanins A and F were assigned by XRD [142,143]. Only compound **424** showed weak antibacterial activity against *Staphylococcus aureus* (MIC = 32 μg/mL vs. 4.0 μg/mL for gentamicin sulfate) [142]. Compounds **420**, **423**, and 3-hydroxypentacecilide A displayed significant antiviral (H1N1) activity (IC_50_ =34–74 μM vs. 103 μM for ribavirin) [142], and compounds **426**, **429,** and **430** exhibited moderate anti-PTP1B activity (IC_50_ = 62.5–71.6 μM) [143].

Indole diterpenoids were the main group of non-polyketide–terpenoids from marine fungi. From the sea anemone-derived fungus *Penicillium* sp. AS-79 (Qingdao, China), 11 indole diterpenoids were isolated, including three new indole diterpenoids, 22-hydroxylshearinine F (**431**), 6-hydroxylpaspalinine (**432**), and 7-*O*-acetylemindole SB (**433**), while only **432** displayed weak activity against *Vibrio parahaemolyticus* (MIC = 64.0 µg/mL) during the antimicrobial tests of 16 strains of pathogenic microbes [144]. A new indole diterpenoid, secopaxilline A (**434**), bearing a rare C–N bond cleavage moiety, was obtained from the marine aciduric fungus *Penicillium camemberti* OUCMDZ-1492 from mangrove soil [14]. Four indole diterpenoids, including the novel penijanthines C and D (**435** and **436**), were explored from the marine sediment-derived fungus *Penicillium janthinellum* (Bohai Sea, China)*,* among which compounds **435** and **436** displayed potent anti-*Vibrio* activity (MIC 3.1–6.30 and 12.5 µM, respectively) against three pathogenic *Vibrio* spp. [145].

When searching for biologically active compounds from the marine fungus *Penicillium* sp. KFD28, which is associated with the bivalve mollusk, *Meretrix lusoria* (Haikou Bay, China), nine new diverse skeletons of indole terpenoids, penerpenes A−D (**437**–**440**) [146] and E–I (**441**–**445**) [147], and nine known indole terpenoids, including paxilline, emindole SB, and 7-hydroxypaxilline-13-ene, were isolated. Notably, compound **437** possesses a 1,4-dihydro-2Hbenzo[d][1,3] oxazine moiety in an unusual spiro indole diterpene; compound **438** has an uncommon heptacyclic scaffold containing pyridine in the indole diterpene; compounds **439**, **440**, and **442** are norindole diterpenoids with new carbon frameworks possibly derived from paxilline via the loss of 3–5 carbons; compound **441** bears a unique 6/5/5/6/6/5/5 heptacyclic ring system; and compound **443** contains an unusual 6/5/5/6/6/7 hexacyclic motif bearing a 1,3-dioxepane ring rarely found in nature [146,147]. Compounds **437** and **438** and emindole SB are considered to be new potent protein tyrosine phosphatase (PTP1B and TCPTP, IC_50_ = 0.7–5.0 μM) inhibitors via PTP inhibition and molecular docking experiments [146]. Compounds **441**, **442**, **444**, and 7-hydroxypaxilline-13-ene inhibited PTP1B (IC_50_ = 13–27 μM). Compound **441** also showed anti-PTPσ activity (IC_50_ = 38 μM), while compound **444** and 7-hydroxypaxilline-13-ene inhibited TCPTP (IC_50_ = 35 and 37 μM, respectively) [147]. Penicindopene A (**446**), possessing a 3-hydroxyl-2-indolone moiety rarely found in indole diterpenes, was isolated from the deep-sea fungus *Penicillium* sp. YPCMAC1 and displayed moderate cytotoxicity (IC_50_ = 15.2 μM for A549, and 20.5 μM for HeLa) [148].

#### 3.6.9. *Pestalotiopsis* sp.

Two new DMOA-derived meroterpenoids, 7-hydroxydehydroaustin (**447**) and 11*β*-acetoxyisoaustinone (**448**), from the seagrass-derived fungus *Pestalotiopsis* sp. PSU-ES 19 were inactive in their antimycobacterial, antimalarial, and cytotoxic activity [149].

#### 3.6.10. *Pleosporales* sp.

The investigation of a marine algae-derived fungus *Pleosporales* sp. led to the isolation of four new merosesquiterpenoids (pleosporallins A–D, **449**–**452**); **449**–**451** displayed moderate anti-inflammatory (IL-6 in RAW264.7) effects, and **452** moderately inhibited the agricultural pathogen *Clavibacter michiganense* subsp. *sepedonicus* (MIC = 9.48 μg/mL) [150].

#### 3.6.11. *Stachybotrys* sp.

A new sulfate meroterpenoid, stachybotrin G (**453**), featuring an atypical farnesylated[1,3]diazepino[2,1-a]isoindole moiety, was isolated from the sponge derived fungus, *Stachybotrys chartarum* (MXH-X73) and lacked bioactivity [151].

Using the OSMAC approach, the marine crinoid-derived fungus, *Stachybotrys chartarum* 952, was able to yield nine metrosesquiterpenes derived from orsellinic acid and farnesyl, including three new linear farnesyl meroterpenoids, stachybonoids A–C (**454**–**456**) and one new phenylspirodrimane, stachybonoid D (**457**), from a solid rice medium, and five phenylspirodrimanes, including two new stachybonoids, E and F (**458**–**459**), from a PDB liquid medium [152]. Compound **454** showed significant inhibition of the dengue virus, and compound **459**, stachybotrysin C, and stachybotrylactone exhibited moderate-to-good anti-inflammatory activity (NO in RAW264.7) (IC_50_ = 17.9–52.5 μM vs. 37.5 μM for indomethacin) [152].

Three structurally diverse phenylspirodrimane-based meroterpenoids with new scaffolds, chartarolides A–C (**460**–**462**), and the known persecutors, stachybotrylactam and mollicellin D, were isolated from the cytotoxic fraction of the sponge-derived fungus, *Stachybotrys chartarum* WGC-25C-6 [153]. Regarding their biogenic synthesis, stachybotrydial with mollicelin J are constituents of the dioxabicyclononane core in **460**–**461**, and stachybotrylactam with mollicellin D combined differently to form **462** [153]. Compounds **460**–**462** showed strong cytotoxicity against six HTCLs (HCT-116, HepG2, BGC-823, NCI-H1650, A2780, and MCF7) (IC_50_ = 1.3–12.5 μM) and exhibited selective significant inhibitory activity towards the human tumor-related protein kinases of FGFR3, IGF1R, PDGFRb, and TrKB (IC_50_ = 2.6–21.4 μM) [153].

#### 3.6.12. *Talaromyces* sp.

The analysis of chemically active components of the mangrove endophytic fungus *Talaromyces amestolkiae* YX1, cultured on a solid wheat substrate medium, resulted in the isolation of four new DMOA-derived meroterpenoids, the amestolkolides A–D (**463**–**466**), which bear a unique and fused polycyclic skeleton with a 6/7/6/5/6 system, along with three known ones, purpurogenolide E and chrodrimanins A and B [154]. Amestolkolides B and A exert potent anti-inflammatory activity by suppressing NO production in LPS-induced RAW264.7 cells (IC_50_ = 1.6 and 30 μM, respectively, vs. 26.3 μM for indomethacin) [154].

Five new unusual polycyclic meroterpenoids, talaromyolides A–D (**467**–**470**), and talaromytin (**471**), were found in the marine fungus *Talaromyces* sp. CX11 [155]. Talaromyolide A (6/6/6/5/5/5 polycyclic skeleton) and talaromyolide D (containing one four-membered ring) are two novel carbon skeletons, and talaromyolides B and C are the first examples of 6/6/6/6/6/6 hexacyclic skeleton meroterpenoids with a seco-drimane sesquiterpene moiety and a C10 polyketide [155]. Talaromytin (**471**), a heptacyclic compound, is found to exist as two slowly interconverting conformers with different side-chain orientations [155]. Additionally, **470** showed potent antiviral (pig PRV) activity with an IC_50_ value of 3.35 μM [155].

## 4. Conclusions

This review provides a comprehensive overview of the diverse chemical structures and bioactive properties of new terpenes that have been isolated from marine-derived fungi in the last five years. There has been a tremendous increase in the rate of new terpenoids being isolated from marine-derived fungi, representing a golden age of microbial-derived compound discovery in the field of marine natural products. Up to 471 new terpenes have been discovered from 133 marine fungal strains belonging to 34 genera. Among them, sesquiterpenes (188, 40%), meroterpenes (165, 35%), and diterpenes (75, 16%) comprise the largest proportion of terpenes, while *Penicillium* (108, 23%), *Aspergillus* (99, 21%), and *Trichoderma* (49, 10%) are the dominant producers of terpenoids. The majority of the fungi producing these novel terpenes were isolated from live marine matter, marine animals (27%), and aquatic plants (including mangrove plants) (38%), while the remaining fungi were obtained from marine environments (i.e., deep-sea sediments (15%), other marine sediments from the shallow sea or coast (11%), and hydrothermal vents (3%)). Around 30% of the undescribed compounds displayed various activities, especially cytotoxicity against HTCLs, which was the case for 54 compounds. Interestingly, some of them with promising or high bioactivities deserve to be paid more attention (shown in Table S1); for example, cytotoxic compounds (chartarenes D (**154**) [46], 14,15-dehydro-6-*epi*-ophiobolin K (**285**), 14,15-dehydro-ophiobolins K (**287**) [84], IC_50_ = 0.14–0.68 μM); lethally toxic compounds (phytoplankton toxicity), trichocarotins C/E (**183/185**) [54], 11*R*-methoxy-5,9,13-proharzitrien-3-ol (**266)** [50], LD_50_ = 0.24–1.3 μg/mL; brine shrimp lethal compounds, 19-hydroxypenitrem A (**349**) [112], LD_50_ = 3.2 μM); antiviral compounds (aperterpenes N (**334**) [107], simpterpenoid (**396)** [128], IC_50_ = 8.1–18 nM); anti-inflammatory compounds (13*β*-hydroxy conidiogenone C (**243**) [67], amestolkolide B (**464**) [154], IC_50_ = 1.6–2.2 μM); enzyme inhibitors (*α*-glucosidase inhibitor, isopimarane diterpene (**239**) [65]; protein tyrosine phosphatases inhibitors, penerpene A (**437**) [146]; human tumor-related protein kinases inhibitors, chartarolide A (**460**) [153], IC_50_ = 1.7–5.0 μM); antibacterial compounds (7-*O*-methylhydroxysydonic acid (**31**) [10], dendry-phiellin I (**53**) [28], penijanthines C (**435**) [145], MIC = 1.5–2.0 μg/mL or 3.1–6.3 μM); and antifungal compounds (wentinoid A (**212**) [60], moriniafungin E (**235**) [64], MIC = 1.0 μg/mL or 2.9 μM). Due to the chemical diversity and biological activities of these terpenoids, it is worth studying marine fungi further to find promising lead compounds for the development of marine drugs.

## Supplementary Materials

The following are available online at https://www.mdpi.com/1660-3397/18/6/321/s1. Table S1: Selective new terpenes with high bioactivities from marine-derived fungi in 2015–2019.
