# Supplementary material for: A Review of Terpenes from Marine-Derived Fungi: 2015–2019
Source: Mar Drugs. 2020 Jun 18;18(6):321. doi: 10.3390/md18060321 (PMC7345631; doi:10.3390/md18060321)
Supplement: Supplementary file 1 [file marinedrugs-18-00321-s001.pdf]

A Review of Terpenes from Marine-Derived Fungi: 2015–2019

Minghua Jiang [1,2,†], Zhenger Wu [1,†], Heng Guo [1], Lan Liu [1,2,3] and Senhua Chen [1,2,3,*]

[1] School of Marine Sciences, Sun Yat-sen University, Guangzhou 510006, China; jiangmh23@mail2.sysu.edu.cn (M.J.); wuzher@mail2.sysu.edu.cn (Z.W.); hengeguo163@163.com (H.G.);
[2] South China Sea Bio-Resource Exploitation and Utilization Collaborative Innovation Center, Guangzhou 510006, China
[3] Southern Laboratory of Ocean Science and Engineering (Guangdong, Zhuhai), Zhuhai 519000, China
* Correspondence: chensenh@mail.sysu.edu.cn; Tel.: +86-020-84725459
† These authors contributed equally to this work.

Table S1: Selective New Terpenes with High Bioactivities from Marine-Derived Fungi in 2015–2019.

Terpenoid name	Structure number	Terpenoid category	Isolate marine fungi	Host/Origin and Location	Bioactivities	Reference
(*R*,*E*)-6-(2,3-dihydroxy-4-methylpenyl)-2-methylhept-5-enoate	**28**	Sesquiterpene	*Aspergillus sydowii* SW9	Sea-water, Yangma Island, China	Antibacterial activity (*Escherichia coli*), MIC= 2.0 μg/ml	21
7-*O*-methylhydroxysydonic acid	**31**	Sesquiterpene	*Aspergillus versicolor* SD-330	Deep-sea-sediment, SCS	Antibacterial activity (*Escherichia coli*), MIC= 2.0 μg/ml	10
14-*O*-acetylinsulicolide A	**33**	Sesquiterpene	*Aspergillus ochraceus* Jcma1F17	Alga *Coelarthrum* sp., SCS	Cytotoxicity vs. ACHN, OS-RC-2 and 786-O, IC_{50}= 4.1, 5.3, 2.3 μM , respectively, arrested the cell cycle in the G0/G1 phase at a concentration of 1 μM and induced late apoptosis at a concentration of 2 μM	23
Asperiene A	**35**	Sesquiterpene	*Aspergillus flavus* CF13-11	Marine sediment, Bohai Sea	Cytotoxicity vs. Hela, MCF-7, MGC-803 and A549, IC_{50}= 3.6, 1.4, 6.8, 5.0 μM	24
Asperiene B	**36**	Sesquiterpene	*Aspergillus flavus* CF13-11	Marine sediment, Bohai Sea	Cytotoxicity vs. Hela, MCF-7, MGC-803, A549 and GES-1, IC_{50}= 2.9, 5.7, 1.9, 2.5, 6.2 μM	24

Asperiene C	**37**	Sesquiterpene	*Aspergillus flavus* CF13-11	Marine sediment, Bohai Sea	Cytotoxicity vs. Hela, MCF-7, MGC-803, A549 and GES-1, IC_{50}= 8.3, 3.1, 7.0, 6.3, 4.9 μM	24
Asperiene D	**38**	Sesquiterpene	*Aspergillus flavus* CF13-11	Marine sediment, Bohai Sea	Cytotoxicity vs. Hela, MCF-7, MGC-803 and A549, IC_{50}= 4.2, 6.6, 2.3, 7.5 μM	24
Dendryphiellin I	**53**	Sesquiterpene	*Cochliobolus lunatus* SCSIO41401	Alga *Coelarthrum* sp., Yongxing Island, SCS	Cytotoxic vs. ACHN, 786-O, OS-RC-2, HepG2 and SGC7901, IC_{50}= 1.4-4.3 μM; antibacterial (*S. Aureus* subsp. Aureus Rosenbach), MIC= 1.5 μg/ml	28
Dendryphiellin J	**54**	Sesquiterpene	*Cochliobolus lunatus* SCSIO41401	Alga *Coelarthrum* sp., Yongxing Island, SCS	Cytotoxicity vs. HepG2 and ACHN, IC_{50} = 5.9, 3.1 μM	28
Diaporol R	**66**	Sesquiterpene	*Diaporthe* sp. IFB-3lp-10	Mangrove plant *Rhizophora stylosa*, Hainan Island, China.	Cytotoxicity vs. SW480, IC_{50} = 8.72 μM	31
Eutyperemophilane I	**77**	Sesquiterpene	*Eutypella* sp. MCCC 3A00281	Deep-sea-sediment, South Atlantic Ocean	Anti-inflammatory activity (NO), IC_{50}= 8.6 μM vs. 16 μM for quercetin	32
Eutyperemophilane J	**78**	Sesquiterpene	*Eutypella* sp. MCCC 3A00281	Deep-sea-sediment, South Atlantic Ocean	Anti-inflammatory activity (NO), IC_{50}= 13 μM vs. 16 μM for quercetin	32
Khusinol B	**100**	Sesquiterpene	*Graphostroma* sp. MCCC 3A00421	Deep-sea hydrothermal sulfide deposit, Atlantic Ocean	Anti-inflammatory activity (NO), IC_{50}= 17 μM vs. 23 μM for aminoguanidine	33
Adametacorenol B	**134**	Sesquiterpene	*Penicillium adametzioides* AS-53	Unidentified marine sponge, Hainan Island, SCS	Cytotoxicity vs. NCI-H446, IC_{50} = 5.0 μM	41
Rhinomilisin A	**138**	Sesquiterpene	*Rhinocladiella similis*	Mangrove fern *Acrostichums aureum* (Pteridaceae), Douala, Cameroon	Cytotoxicity vs. L5178Y, IC_{50} = 5.0 μM	43
Rhinomilisin G	**144**	Sesquiterpene	*Rhinocladiella similis*	Mangrove fern *Acrostichums aureum* (Pteridaceae), Douala, Cameroon	Cytotoxicity vs. L5178Y, IC_{50} =8.7 μM	43
Chartarene A	**151**	Sesquiterpene	*Stachybotrys chartarum* WGC-25C-6	Sponge *Niphatesrecondite*, Beibuwan Bay, SCS	Cytotoxicity vs. HCT-116, hepg2, BGC-823, and A2780, IC_{50}= 2.38-3.95 μM	46
Chartarene B	**152**	Sesquiterpene	*Stachybotrys chartarum* WGC-25C-6	Sponge *Niphatesrecondite*, Beibuwan Bay, SCS	Cytotoxicity vs. HCT-116, IC_{50}= 5.58 μM	46

Chartarene C	**153**	Sesquiterpene	*Stachybotrys chartarum* WGC-25C-6	Sponge *Niphatesrecondite*, Beibuwan Bay, SCS	Cytotoxicity vs. HCT-116, hepg2, NCI-H1650 and A2780, IC_{50}= 0.74-2.58 μM	46
Chartarene D	**154**	Sesquiterpene	*Stachybotrys chartarum* WGC-25C-6	Sponge *Niphatesrecondite*, Beibuwan Bay, SCS	Cytotoxicity vs. HCT-116, hepg2, BGC-823, NCI-H1650 and A2780, IC50= 0.68-1.49 μM	46
11-Methoxy-9-cycloneren-3,7-diol	**163**	Sesquiterpene	*Trichoderma harzianum* X5	Brown alga *Laminaria japonica,* Chang Islands, China	Phytoplankton lethality–toxicity of *C. Marina* and *K. Veneficum*, IC_{50} = 0.66, 2.2 μg/ml	50
4-Cadinen-11,12-diol	**169**	Sesquiterpene	*Trichoderma asperellum* A-YMD-9-2	Red alga *Gracilaria verrucosa*, Yangma Island, China	Phytoplankton lethality–toxicity of *C. Marina*, *H. Akashiwo* and *K. Veneficum*, IC_{50} = 1.1-2.1 μg/ml	52
Trichobisabolin B	**174**	Sesquiterpene	*Trichoderma asperellum* Y6–2	Red alga *Chondrus ocellatus*	Phytoplankton lethality–toxicity of *H. Akashiwo*, IC_{50} = 2.2 μg/ml	53
Trichobisabolin H	**180**	Sesquiterpene	*Trichoderma asperellum* Y6–2	Red alga *Chondrus ocellatus*	Phytoplankton lethality–toxicity of *H. Akashiwo*, *P. Donghaiense*, *C. Marina*, and *K. Veneficum*, IC_{50}=1.9-3.8 μg/ml	53
Trichocarotin C	**183**	Sesquiterpene	*Trichoderma virens* von Arx Y13-3 (Moniliaceae)	Red alga *Gracilaria vermiculophylla* (Ohmi) Papenfuss (Gracilariaceae), Yangma Island, China	Phytoplankton lethality–toxicity of *C. Marina*, IC_{50} = 0.24 μg/ml	54
Trichocarotin D	**184**	Sesquiterpene	*Trichoderma virens* von Arx Y13-3 (Moniliaceae)	Red alga *Gracilaria vermiculophylla* (Ohmi) Papenfuss (Gracilariaceae), Yangma Island, China	Phytoplankton lethality–toxicity of *C. Marina*, IC_{50} = 0.33 μg/ml	54
Trichocarotin E	**185**	Sesquiterpene	*Trichoderma virens* von Arx Y13-3 (Moniliaceae)	Red alga *Gracilaria vermiculophylla* (Ohmi) Papenfuss (Gracilariaceae), Yangma Island, China	Phytoplankton lethality–toxicity of *C. Marina*, IC_{50} = 0.27 μg/ml	54
Trichocarotin H	**188**	Sesquiterpene	*Trichoderma virens* von Arx Y13-3 (Moniliaceae)	Red alga *Gracilaria vermiculophylla* (Ohmi) Papenfuss (Gracilariaceae), Yangma Island, China	Phytoplankton lethality–toxicity of *C. Marina*, IC_{50} = 1.2 μg/ml	54
Virescenoside Z_{10}	**201**	Diterpene	*Acremonium striatisporum* KMM4401	Holothurian *Eupentacta fraudatrix*	Anti-inflammatory activity (ROS) in the macrophages by 45% at a concentration of 10 μM, anti-inflammatory activity (NO) at a concentration of 1 μM	58

Virescenoside Z_{13}	**204**	Diterpene	*Acremonium striatisporum* KMM4401	Holothurian *Eupentacta fraudatrix*	Anti-inflammatory activity (NO) at a concentration of 1 μM	58
Asperolide E	**211**	Diterpene	*Aspergillus wentii* SD-310	Deep-sea-sediment, SCS	Cytotoxicity. Hela, MCF-7, and NCI-H446, IC_{50}= 10.0, 11.0, 16.0 μM	59
Wentinoid A	**212**	Diterpene	*Aspergillus wentii* SD-310	Deep-sea-sediment, SCS	Plant pathogenic antifungal activity (*P. Parasitica*, *F. Oxysporum* f. Sp. Lycopersici, *F. Graminearum* and *B. Dothidea)*, MIC=8.0, 4.0, 1.0, 4.0 μg/ml	60
Moriniafungin E	**235**	Diterpene	*Curvularia hawaiiensis* TA26-15	Zoanthid *Palythoa haddoni*, Weizhou coral reefs, SCS	Antifungal activity (*C. Albicans* ATCC10231), MIC= 2.9 μM	64
Isopimarane diterpene	**239**	Diterpene	*Epicoccum* sp. HS-1	*Apostichopus japonicus*	α-glucosidase inhibitor activity, IC_{50}= 4.6 μM vs. 31.2 μM for resveratrol	65
13β-hydroxy conidiogenone C	**243**	Diterpene	*Penicillium* sp. TJ403-2	Sea sediment	Anti-inflammatory activity (NO), IC_{50}= 2.19 μM vs. 8.76 μM three-fold lower than indomethacin	67
Roussoellol C	**251**	Diterpene	*Talaromyces purpurogenus* PP-414	Mud sample, coastal beach in Qinghuangdao County, China	Cytotoxicity vs. MCF-7 and HL-60, IC_{50}= 6.5, 10.9 μM	47
11*R*-methoxy-5,9,13-proharzitrien-3-ol	**266**	Diterpene	*Trichoderma harzianum* X5	Brown alga *Laminaria japonica*, Chang Islands, China	Phytoplankton lethality–toxicity of *H. Akashiwo* and *P. Donghaiense*, IC_{50}= 1.2-1.3 μg/ml	50
Trichodermanin C	**267**	Diterpene	*Trichoderma harzianum* OUPS-111D-4	Sponge *Halichondria okadai*	Cytotoxicity vs. P388, HL-60 and L1210, IC_{50}= 6.8-7.9 μM	76
Ophiobolin Z	**280**	Sesterterpene	*Aspergillus ustus* 094102	Rhizosphere soil of mangrove *Bruguiera gymnorrhiza*， SCS	Cytotoxicity vs. MCF/Adr, IC_{50}=5.4 μM	83
21-*epi*-Ophiobolin Z	**282**	Sesterterpene	*Aspergillus ustus* 094102	Rhizosphere soil of mangrove *Bruguiera gymnorrhiza*， SCS	Cytotoxicity vs. MCF-7 MCF/Adr, IC_{50}=7.9-9.4 μM	83
21-*epi*-Ophiobolin O	**283**	Sesterterpene	*Aspergillus ustus* 094102	Rhizosphere soil of mangrove *Bruguiera gymnorrhiza*， SCS	Cytotoxicity vs. A549, and HL-60, IC_{50}=0.6-0.8 μM	83
14,15-dehydro-6-*epi*-ophiobolin K	**285**	Sesterterpene	*Aspergillus flocculosus*	Algae *Padina* sp, Da Nang, Vietnam	Cytotoxicity vs. HCT-15, NUGC-3, NCI-H23, ACHN, PC-3, and MDA-MB-231, IC_{50}= 0.14-0.24 μM	84

14,15-dehydro-ophiobolin K	**287**	Sesterterpene	*Aspergillus flocculosus*	Algae *Padina* sp, Da Nang, Vietnam	Cytotoxicity vs. HCT-15, NUGC-3, NCI-H23, ACHN, PC-3, and MDA-MB-231, IC_{50}= 0.44-0.63 μM	84
14,15-dehydro-6-*epi*-ophiobolin G	**286**	Sesterterpene	*Aspergillus flocculosus*	Algae *Padina* sp, Da Nang, Vietnam	Cytotoxicity vs. HCT-15, NUGC-3, NCI-H23, ACHN, PC-3, and MDA-MB-231, IC_{50}= 0.88-1.14 μM	84
14,15-dehydro-ophiobolin G	**288**	Sesterterpene	*Aspergillus flocculosus*	Algae *Padina* sp, Da Nang, Vietnam	Cytotoxicity vs. HCT-15, NUGC-3, NCI-H23, ACHN, PC-3, and MDA-MB-231, IC_{50}= 1.07-1.50 μM	84
14,15-dehydro-(*Z*)-14-ophiobolin G	**289**	Sesterterpene	*Aspergillus flocculosus*	Algae *Padina* sp, Da Nang, Vietnam	Cytotoxicity vs. HCT-15, NUGC-3, NCI-H23, ACHN, PC-3, and MDA-MB-231, IC_{50}= 1.53-2.01 μM	84
Asperophiobolin H	***297***	Sesterterpene	*Aspergillus* sp. ZJ-68	Mangrove plant *Kandelia candel*, Zhanjiang Mangrove Nature Reserve, SCS	Anti-inflammatory activity (NO) (IC_{50}= 21 μM vs. 38 μM for indomethacin), anti-MPTPB activity (IC_{50} =19 μM vs. 22 μM for oleanolic acid)	85
Asperophiobolin I	***298***	Sesterterpene	*Aspergillus* sp. ZJ-68	Mangrove plant *Kandelia candel*, Zhanjiang Mangrove Nature Reserve, SCS	Anti-inflammatory activity (NO) (IC_{50} = 24 μM vs. 38 μM for indomethacin)	85
Asperophiobolin J	**299**	Sesterterpene	*Aspergillus* sp. ZJ-68	Mangrove plant *Kandelia candel*, Zhanjiang Mangrove Nature Reserve, SCS	Anti-inflammatory activity (NO) (IC_{50} = 26 μM vs. 38 μM for indomethacin)	85
Austalide S	**318**	Meroterpene	*Aspergillus aureolatus* HDN14-107	Unidentified sponge, Xisha Island, SCS	Anti-virus (H1N1), IC_{50} = 90 μM vs. 102 μM for ribavirin)	98
Aperterpenes N	***334***	Meroterpene	*Aspergillus terreus* EN-539	Marine red alga *Laurencia okamurai,* coast of Qingdao, China	Anti-virus (influenza neuraminidase) (IC_{50} = 18.0 nM vs. 3.2 nM for oseltamivir)	107
Tricycloalternarene 14b	**340**	Meroterpene	*Aspergillus* sp. D	Coastal plant *Edgeworthia chrysantha* Lindl., Hangzhou Bay, China	Cytotoxicity vs. A-549 cells (IC_{50} = 8.89 μM)	109
19-hydroxypenitrem A	**349**	Meroterpene	*Aspergillus nidulans* EN-330	Marine red alga *P. Scopulorum* var. Villum, Yantai coastline, China	Brine shrimp cytotoxic activity (LD_{50} = 3.2 μM vs. 10.7 μM for colchicine)	112
19-hydroxypenitrem E	**350**	Meroterpene	*Aspergillus nidulans* EN-330	Marine red alga *P. Scopulorum* var. Villum, Yantai coastline, China	Brine shrimp cytotoxic activity (LD_{50} = 4.6 μM vs. 10.7 μM for colchicine)	112

Eupeniacetal A	**351**	Meroterpene	*Eupenicillium* sp. 6A-9	Sponge *Plakortis simplex*, Xisha Island, SCS	Immune-suppressive activity (TNF-α) (IC$_{50}$ = 22.6μM vs. 0.23 μM for pomalidomide)	113
Hydroberkeleyone B	**354**	Meroterpene	*Eupenicillium* sp. 6A-9	Sponge *Plakortis simplex*, Xisha Island, SCS	Immune-suppressive activity(TNF-α) ((IC$_{50}$ = 28.5μM vs. 0.23 μM for pomalidomide)	113
Penicilindole B	***356***	Meroterpene	*Eupenicillium* sp. HJ002	Mangrove *Xylocarpus granatum* Koenig, SCS	Cytotoxicity vs. A549 and hepg2 (IC$_{50}$ = 5.5 and 1.5 μM)	114
Rhizovarin A	**360**	Meroterpene	*Mucor irregularis* QEN-189	Mangrove plant *Rhizophora stylosa*, Hainan Island, SCS	Cytotoxicity vs. A-549 and HL-60 (IC$_{50}$ = 9.6–11.5 μM)	116
Rhizovarin B	**361**	Meroterpene	*Mucor irregularis* QEN-189	Mangrove plant *Rhizophora stylosa*, Hainan Island, SCS	Cytotoxicity vs. A-549 and HL-60 (IC$_{50}$ =5.0-6.3 μM)	116
Rhizovarin E	**364**	Meroterpene	*Mucor irregularis* QEN-189	Mangrove plant *Rhizophora stylosa*, Hainan Island, SCS	Cytotoxicity vs. A-549 (IC$_{50}$ = 9.2 μM),	116
Brasilianoid A	**374**	Meroterpene	*Penicillium brasilianum* WZXY-m122-9	Unidentified sponge，Beibu Gulf, SCS	Promoted the expression of filaggrin and caspase-14 in hacat cells in a dose-dependent manner	121
Brasilianoid L	**385**	Meroterpene	*Penicillium brasilianum* WZXY-m122-9	Unidentified sponge，Beibu Gulf, SCS	Inhibit bacteria-infected host cells by preventing the polymerization of actin in RAW264.7	122
Andrastone A	**394**	Meroterpene	*Penicillium allii-sativi*	Deep-sea Water, western Pacific	Cytotoxicity vs. HepG2 (IC$_{50}$ = 7.8 μM)	127
Simpterpenoid	**396**	Meroterpene	*Penicillium simplicissimum* MA-332	Rhizospheric soil of mangrove plant *Bruguiera sexangula*	Anti-virus (influenza neuraminidase) (IC$_{50}$ = 8.1 nM vs. 3.2 nM for oseltamivir)	128
Penicilliumin B	**417**	Meroterpene	*Penicillium* sp. F00120	Deep-sea-sediment, SCS	Inhibit the kidney fibrogenic action	140
Verruculide A	**418**	Meroterpene	*Penicillium verruculosum* TPU1311	Ascidian,Chordata, *Polycarpa aurat*，Manado, Indonesia,	Anti-PTP1B activity (IC$_{50}$ = 8.4–14.9 μM),	141
ChrodrimaninK	***420***	Meroterpene	*Penicillium* sp. SCS-KFD09	Marine worm, *Sipunculus nudus*, Haikou Bay, SCS	Antivirus (H1N1) (IC$_{50}$ =34 μM vs. 103 μM for ribavirin)	142
Chrodrimanin N	**423**	Meroterpene	*Penicillium* sp. SCS-KFD09	Marine worm, *Sipunculus nudus*, Haikou Bay, SCS	Antivirus (H1N1) (IC$_{50}$ =74 μM vs. 103 μM for ribavirin)	142
Penijanthine C	**435**	Meroterpene	*Penicillium camemberti* OUCMDZ-1492	Mangrove soil, Wenchang mangrove natural reserve area, SCS	Antibacterial (*Vibrio*) activity (MIC 3.1–6.30 μM)	145

Penerpene A	**437**	Meroterpene	*Penicillium sp.* KFD28	Bivalve mollusk, *Meretrix lusoria*, Haikou Bay, SCS	Protein tyrosine phosphatases inhibitors (PTP1B and TCPTP, IC_{50} = 1.7–5.0 μM)	146
Penerpenes B	**438**	Meroterpene	*Penicillium sp.* KFD28	Bivalve mollusk, *Meretrix lusoria*, Haikou Bay, SCS	Protein tyrosine phosphatases inhibitors (PTP1B and TCPTP, IC_{50} = 2.4-4.5 μM)	146
ChartarolideA	**460**	Meroterpene	*Stachybotrys chartarum* WGC-25C-6	Sponge *Niphatesrecondite*, Beibuwan Bay, SCS	Cytotoxicity vs. HCT-116, HepG2, BGC-823, NCI-H1650, A2780, and MCF7)(IC_{50} = 1.3–5.5 μM) and human tumor-related protein kinases inhibitors of FGFR3, IGF1R, pdgfrb, and trkb (IC_{50} = 2.6–9.1 μM)	153
Chartarolide B	**461**	Meroterpene	*Stachybotrys chartarum* WGC-25C-6	Sponge *Niphatesrecondite*, Beibuwan Bay, SCS	Cytotoxicity vs. HCT-116, hepg2, BGC-823, NCI-H1650, A2780, and MCF7 (IC_{50} = 1.6–3.8 μM) and human tumor-related protein kinases inhibitors of FGFR3 and IGF1R (IC_{50} = 4.9–8.4 μM)	153
Chartarolide C	**462**	Meroterpene	*Stachybotrys chartarum* WGC-25C-6	Sponge *Niphatesrecondite*, Beibuwan Bay, China	Cytotoxicity vs. HCT-116, HepG2, BGC-823, and MCF7 (IC_{50} = 5.4–8.9 μM)	153
Amestolkolide B	**464**	Meroterpene	*Talaromyces amestolkiae* YX1	Mangrove *Kandelia obovata*, Zhanjiang Mangrove Nature Reserve, SCS	Anti-inflammatory activity (NO) (IC_{50} =1.6 μM, vs. 26.3 μM for indomethacin)	154
Talaromyolide D	**470**	Meroterpene	*Talaromyces* sp. CX11	Unreported	Antivirus (pig PRV) , IC_{50} = 3.35 μM	155

PS: The new terpenes from marine fungi were selected by their high bioactivities, such as their activities with IC_{50}/MIC≤10μM or MIC≤3μg/ml, or better than/ compared with the selected positive control. SCS=South China Sea, cytotoxicity = cytotoxicity against tumor cell lines (TCLs), mainly human TCLs,
